# A facile one-pot synthesis of tetrahydrobenzo[*b*]pyrans and 2-amino-4*H*-chromenes under green conditions†

**DOI:** 10.1039/d4ra04239e

**Published:** 2024-07-16

**Authors:** Sarieh Momeni, Ramin Ghorbani-Vaghei

**Affiliations:** a Department of Organic Chemistry, Faculty of Chemistry and Petroleum Sciences, Bu-Ali Sina University Hamedan Iran rgvaghei@yahoo.com ghorbani@basu.ac.ir

## Abstract

This research developed a new nanocatalyst by incorporating nanocopper iodide onto the surface of a layered double hydroxides modified. This new nanocatalyst enables the green synthesis of tetrahydrobenzo[*b*]pyrans and 2-amino-4*H*-chromene derivatives through a one-pot, three-component reaction, demonstrating remarkable activity and selectivity. Key advantages of this method include increased products yield (86–96%), rapid reaction kinetics (5–23 minutes), low reaction temperature (40 °C), synthesis of new products, straightforward purification methods, catalyst recyclability (up to 4 cycles), and solvent-free conditions.

## Abstract 2

This research developed a new nanocatalyst by incorporating nanocopper iodide onto the surface of modified layered double hydroxides.

## Graphical abstract

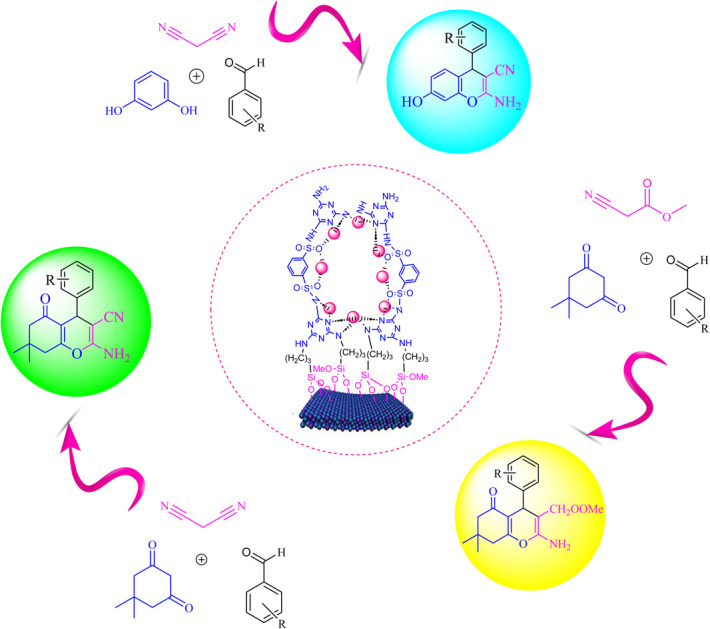

## 1. Introduction

Layered double hydroxides (LDHs), a widely studied class of inorganic layered materials, exhibit remarkable capability for anion intercalation. They possess two-dimensional nanostructures described by the formula [M_(1−*x*)_^2+^M_*x*_^3+^(OH)_2_](A^*n*−^)_*x*/*n*_·*z*H_2_O], where M^2+^ and M^3+^ represent divalent and trivalent metal ions, respectively. These metal ions, including Zn^2+^, Mg^2+^, Ca^2+^, Mn^2+^, Co^2+^, Fe^2+^, Al^3+^, Cr^3+^, Fe^3+^, Co^3+^, and Mn^3+^, replace each other interchangeably in the structure. The interlayer anions, often chloride, bromide, carbonate, or nitrate, along with the molar ratio *x* (ranging from 0.20 to 0.33), play a crucial role in preventing unfavorable phase formation.^1–3^ LDHs possessing abundant basic sites within their brucite-like layers serve as effective heterogeneous solid base catalysts, finding extensive applications in organic synthesis, including but not limited to Michael addition, aldol and Claisen–Schmidt condensation, and Knoevenagel condensation. LDH nanostructures show promise across various fields like adsorption, photochemistry, catalysis, molecular recognition, and catalysis.^4–6^ Particularly in biomedicine, their layered structure and ability for anionic exchange make them significant. Notably, LDH nanoparticles excel in drug delivery, allowing for encapsulation of specific compounds through interlayer anion exchange.^7^

Nanomaterials have revolutionized catalytic chemistry, offering unprecedented opportunities for enhancing reaction efficiency and selectivity while minimizing environmental impact. These materials, engineered at the nanoscale, possess unique properties such as high surface area-to-volume ratio, tunable surface chemistry, and size-dependent reactivity, making them ideal candidates for catalytic applications.^8,9^

The development of materials through chemical synthesis is evolving with a fresh perspective on environmental stewardship. Researchers in this domain are progressively incorporating eco-conscious techniques, such as employing non-toxic solvents, conducting reactions without solvents, employing recyclable catalysts, and embracing one-pot multicomponent reactions. These methods are aimed at mitigating the environmental effects of chemical processes.^10,11^

Multi-component reactions (MCRs) have emerged and expanded as a potent strategy for synthesizing a broad array of drug-like compounds with diverse structures. Key benefits of this approach include high yields, shortened reaction times, enhanced atomic economy, and reductions in both solvent usage and energy consumption.^12,13^

In recent years, nitrogen-linked heterocyclic compounds have garnered significant interest owing to their extensive utility across various applications.^14,15^ Tetrahydrobenzo[*b*]pyrans, also referred to as tetrahydro-4*H*-chromenes, are highly esteemed fused oxygen-containing heterocycles appreciated for their diverse applications in synthetic organic and medicinal chemistry.^16–18^ They encompass a wide range of biological activities, including anticancer,^19^ anti-coagulant,^20^ anti-HIV,^21^ antibacterial,^22^ antiviral,^23^ anti-inflammatory,^24^ diuretic, antihypertensive,^25^ antioxidant.^14^ Notably, several of these heterocycles hold promise in the treatment of conditions such as Schizophrenia, Alzheimer's, amyotrophic lateral sclerosis, Huntington's, Parkinson's, and Down's syndrome. These reactions employ a variety of catalytic systems, such as CeCl_3_·7H_2_O,^26^ Fe_3_O_4_@SiO_2_@NiSB,^27^, [DABCO-PDO][CH_3_COO],^28^ Co_3_(PO_4_)_2_,^29^ NH_2_@SiO_2_@Fe_3_O_4_ MNPs,^30^ f rGO@Fe_3_O_4_@ZrCp_2_Cl_2_ ionic liquid [PEMIM][OH],^31^ and Nano-SiO_2_/DBN.^32^ Moreover, alternative synthesis conditions like electrochemical conditions and microwave radiation have also been documented.

The chromene ring system has garnered significant interest in pharmaceutical research due to the diverse biological properties observed in synthetic and natural chromene derivatives, such as antibacterial,^33^ antimicrobial,^34^ anti-inflammatory,^35^ antiproliferative, antifungal,^36^ and anticancer activities.^37^ Notably, the synthesis of 2-amino-4*H*-chromenes and their derivatives *via* multi-component reactions (MCRs) involving aromatic aldehydes, resorcinol, and malononitrile has been explored. Various catalyst systems have been employed for this synthesis, such as tungstic acid-SBA-15,^38^ Fe_3_O_4_-chitosan nanoparticles,^39^ CuO–CeO_2_,^40^ nano-sized MgO,^41^ sulfonic acid-functionalized MIL-101(Cr),^42^ potassium phthalimide-*N*-oxyl (POPINO),^43^ K_2_CO_3_,^44^ Na_2_CO_3_,^45^ and Mg/Al-HT.^46^

Yet, given the sustained interest in this domain, there remains a call for research into uncomplicated methodologies, reduced reaction times, and catalysts that are both more efficient and reusable, to meet the ongoing demand for the synthesis of 2-amino-4*H*-chromenes and tetrahydrobenzo[*b*]pyrans derivatives in one-pot reactions.

Continuing our pursuit of advancing eco-friendly heterogeneous nanocatalysts and their utilization in catalyzing organic reactions and synthesizing heterocyclic compounds, we present the synthesis and characterization of CuI stabilized on LDH functionalized with a sulfonamide ligand. LDH@PTRMS@NDBD@CuI served as a highly effective and reusable heterogeneous nanocatalyst in facilitating the three-component one-pot synthesis of 4*H*-chromene (4a–h) and tetrahydrochromene.

## 2. Experimental

### 2.1. General

In this study, chemicals were obtained from Merck and used without additional purification. Characterization methods included FT-IR spectroscopy with a PerkinElmer GX FT-IR spectrometer, 1H and ^13^C NMR spectroscopy in DMSO-d_6_ using Bruker BioSpin GmbH 300 MHz FT NMR spectrometers, and melting point determination using a BUCHI 510 device. The novel LDH@PTRMS@NDBD@CuI catalyst was thoroughly characterized through FTIR, EDX, mapping, XRD, FESEM, TGA, and DSC analyses. XRD patterns were taken with a Philips PW1730 in the range of 10 to 90° (2*θ*), and FESEM analysis was performed using a FE-SEM TESCAN MIRA3 instrument. EDX analysis was conducted with EDAX-EDS equipment, TGA was performed using a TGA-DTA instrument in a nitrogen atmosphere, and DSC measurements were taken with a DSC device. The progress of reactions and material purity were monitored using thin layer chromatography (TLC) and silica gel plates.

### 2.2. Protocol for synthesizing layered double hydroxides (Zn/Cr-LDHs)

The synthesis of Zn/Cr-LDH followed the previously described procedure, outlined as follows: initially, Zn(NO_3_)_2_·6H_2_O and Cr(NO_3_)_3_·9H_2_O salts were dissolved in deionized water in a 2 : 1 molar ratio. The solution's pH was subsequently raised to 11.5 by titrating with a 2 M NaOH aqueous solution while vigorously stirring, and this pH level was upheld for a duration of 18 hours at constant temperature. After filtration, the resulting turquoise product incurred washing with distilled water, followed by drying in an oven at 50 °C for 24 hours.^6^

### 2.3. Method for synthesizing LDHs coated with 3-chloropropyltrimethoxysilane (Zn/Cr-LDH@PTRMS)

To activate LDH, we employed 3-chloropropyltrimethoxysilane (PTRMS). Initially, 1 g of pre-synthesized LDH was dispersed within a 100 mL balloon containing 50 mL of toluene. Next, 2 mL of PTRMS was introduced into the solution, which was refluxed for 24 h with continuous stirring. Following refluxing, a precipitate formed, which was collected using filter paper. Subsequently, the precipitate incurred multiple washes with toluene and ethanol to eliminate impurities. Finally, the washed material was dried in an oven at 50 °C to ensure the complete removal of solvents and moisture.^6^

### 2.4. Method for synthesizing ligand of *N*_1_,*N*_4_-bis(4,6-diamino-1,3,5-triazin-2-yl)benzene-1,4-disulfonamide (NDBD)

To begin, phosphorus pentachloride, acting as the chlorinating reagent, was introduced, totaling 16.5 mmol, into a vessel containing 5.00 g (18 mmol) of 1,3-benzenedisulfonic acid disodium salt. The mixture was then heated to 65 °C, allowing the reaction to proceed for 2 hours. Following completion, a solution composed of 150 g of dry ice and 150 mL of chloroform was added, facilitating the separation of the organic layer containing 1,3-benzenedisulfonyl chloride.

Following the separation, 1,3-benzenedisulfonyl chloride (2 mmol) was dissolved in 30 mL of toluene solvent in a 50 mL round-bottom flask. Concurrently, melamine (4 mmol) was dissolved in toluene within a distinct vessel, and this melamine–toluene solution was carefully introduced drop by drop into the flask. Subsequently, the flask was refluxed for a duration of 24 hours. Following this, the resultant precipitate was filtered and subjected to multiple washes with acetonitrile (3 mL each). Finally, the product was dried at 60 °C for a period of 18 hours.^2^

### 2.5. Method for synthesizing LDH@PTRMS@NDBD

LDH@TRMS (0.3 g) was introduced into a 250 mL round-bottom flask containing toluene (80 mL) as the solvent. The mixture incurred ultrasonication for 30 minutes. Next, the ligand NDBD (0.55 g) was incorporated, and the solution was stirred under reflux conditions for 48 hours. Afterward, the resulting mixture was filtered and subjected to several washes with toluene (2 mL) and one wash with ethanol (2 mL). Lastly, the product was dried in an oven at 60 °C for 24 hours.^2^

### 2.6. Method for synthesizing copper iodide (CuI) nanoparticles

CuSO_4_ (1 mmol) was ultrasonically treated in acetone, followed by a HCl (2 M) solution, and then rinsed several times with distilled water. After drying, the copper sulfate was added to a solution of KI (2 mmol) in 40 mL of distilled water and subjected to ultrasound for 30 minutes. The resulting purple precipitate was separated by centrifugation, washed with distilled water and ethanol, and then dried in an oven at 60 °C for 24 hours.

### 2.7. Method for synthesizing LDH@PTRMS@NDBD@CuI

**Scheme 1:**
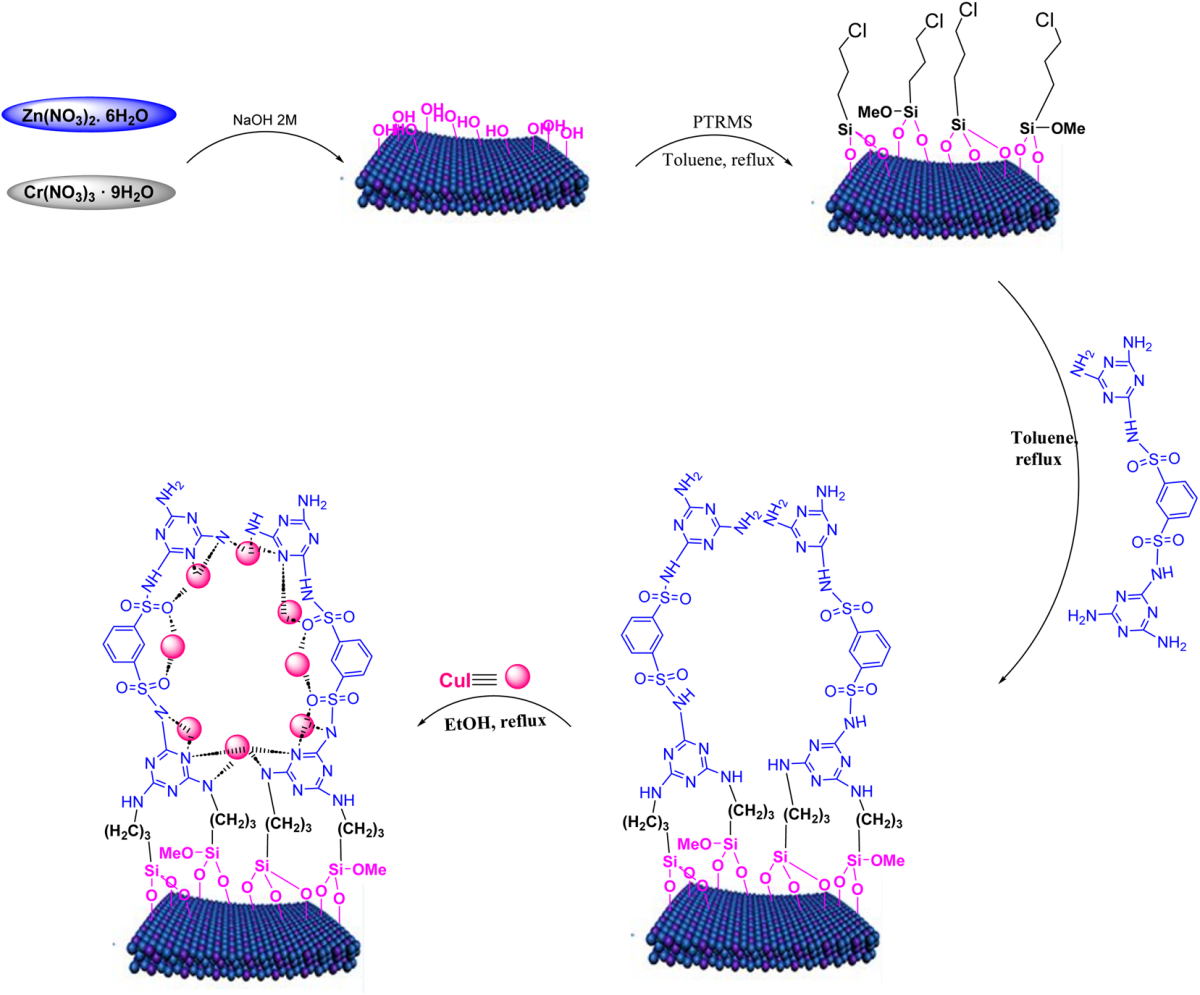
The steps of catalytic LDH@PTRMS@NDBD@CuI synthesis.

In a 25 mL flask, 0.3 g of copper iodide nanoparticles were combined with 0.5 g of LDH@TRMS@NDBD powder, and dissolved in 20 mL of ethanol. The resulting reaction mixture was reflux for 24 hours while being stirred with a magnetic stirrer. Subsequently, LDH@PTRMS@NDBD@CuI nanoparticles were obtained through centrifugation, followed by three washes with ethanol (4 mL each). Finally, the nanoparticles were dried in an oven at 60 °C, the steps of catalytic LDH@PTRMS@NDBD@CuI synthesis were illustrated in Scheme 1.

### 2.8. Method for synthesizing tetrahydrobenzo[*b*]pyrans and 2-amino-4*H*-chromenes derivatives in the presence of LDH@PTRMS@NDBD@CuI nanoparticles

Malononitrile (1 mmol), along with dimedone or resorcinol (1 mmol) and various derivatives of aromatic aldehydes (1 mmol), were combined with LDH@PTRMS@NDBD@CuI nanoparticles (0.05 g) under solvent-free conditions at 40 °C. The mixture was blended in a test tube and stirred for an appropriate duration (5 to 23 minutes) using a magnetic stirrer. The progression of the reaction was monitored using TLC (ethyl acetate/normal hexane: 4/8). Once the reaction reached its conclusion and the desired compound was formed, the mixture was cooled until it reached the room temperature. To separate the catalyst, 3 mL of hot ethanol or ethyl acetate was added to the mixture, followed by stirring for 2 minutes. Following that, the catalyst (LDH@PTRMS@NDBD@CuI) was separated through centrifugation, subjected to washing, and subsequently dried in an oven set at 60 °C.

The solvent within the reaction mixture was evaporated, and the resultant products were dissolved in ethanol using nebulization. The synthesis of the compounds was accomplished with remarkable efficiency, as illustrated in Table 5.

FTIR, ^1^H NMR, and ^13^C NMR spectra were utilized to identify the products, with the melting points of all products being determined.

## 3. Results and discussions

### 3.1. Spectroscopic investigation to identify nanocopper catalyst stabilized on double hydroxide layer coated with ligand of NDBD

**Fig. 1:**
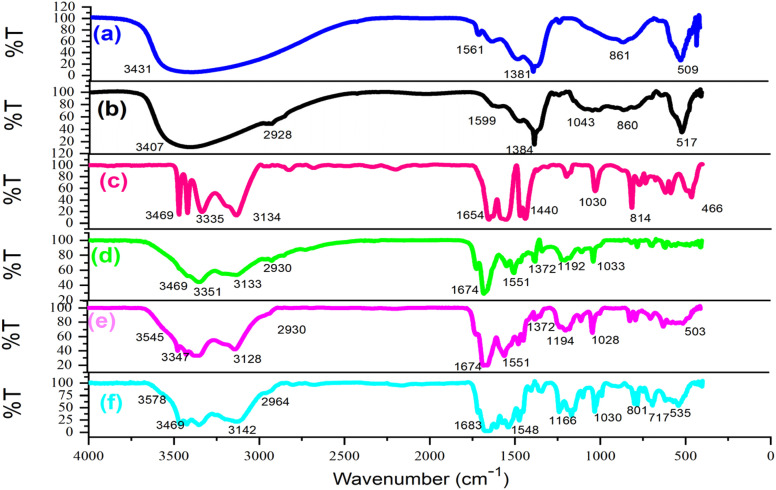
FTIR spectra of (a) double hydroxide layer, (b) LDH@PTRMS, (c) melamine (d) NDBD ligand, (e) LDH@PTRMS@NDBD and (f) LDH@PTRMS@NDBD@CuI.

Following the synthesis of the catalyst, a comprehensive array of analyses such as Fourier-transform infrared spectroscopy (FT-IR), field emission scanning electron microscopy (FESEM), X-ray dispersive analysis (EDX), elemental imaging, X-ray diffraction (XRD), and thermal decomposition (DSC, TGA) were conducted to confirm and characterize the LDH@PTRMS@NDBD@Cu catalyst. Fig. 1 illustrates the FT-IR spectrum of (a) the double hydroxide layer, (b) LDH@PTRMS, (c) melamine, (d) the ligand (NDBD), (e) LDH@PTRMS@NDBD, and a portion of LDH@PTRMS@NDBD@CuI is represented by (f).

Section (a) depicts LDH, characterized by peaks ranging from 2500 to 3490 cm^−1^ attributed to the hydroxides present on LDH, while those within the range of 1380–1400 cm^−1^ signify the stretching vibrations of the interlayer nitrate anion. Furthermore, the peak observed at 853 cm^−1^ corresponds to metal–oxygen stretching vibrations.^47^ In contrast, section (b) illustrates LDH functionalized with 3-chloropropyltrimethoxysilane, as indicated by the peak at 2800 cm^−1^, which signifies C–H stretching vibrations.^47^

In segment (c) of the spectrum, which corresponds to melamine, peaks associated with NH are evident within the range of 3133–3470 cm^−1^, while stretching vibrations of the ring cyanide are apparent at 1652 cm^−1^ and 1631 cm^−1^.^2^ Moving to segment (d) attributed to the NDBD ligand, peaks within the 3180–3410 cm^−1^ range correspond to NH stretching vibrations.^2^ Additionally, the peak at 1722 cm^−1^, previously associated with ring cyanide stretching vibrations at 1652 cm^−1^, has shifted due to the formation of the amide group near the ring C=N. Another peak at 1679 cm^−1^, originally from the region 1631 cm^−1^, is also linked to ring cyanide vibrations. Moreover, the peaks observed at 1361 and 1144 cm^−1^ are attributable to the stretching vibrations of SO. In segment (e), where the O–H peaks are concealed and the ligand is introduced, vibrations associated with NH (3110–3410 cm^−1^) and SO (1357 and 1141 cm^−1^) are detected. Furthermore, the appearance of the peak at 2956 cm^−1^ aligns with the CH stretching vibrations of the PTRMS, validating the complete bonding of the ligand to LDH.^2^

In the final step (f), notable reductions in the stretching vibrations of NH and sulfonyl amide groups are observed, while peaks at 517, 574, and 1384 cm^−1^ are indicative of copper iodide presence, confirming its successful immobilization onto the catalyst.

**Fig. 2:**
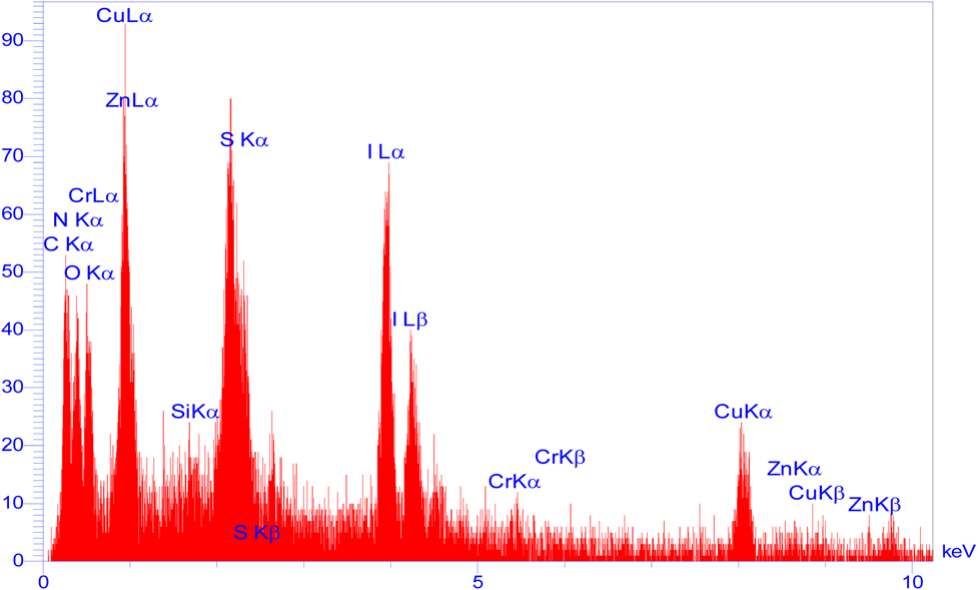
EDX analysis of LDH@PTRMS@NDBD@CuI catalyst.

**Fig. 3:**
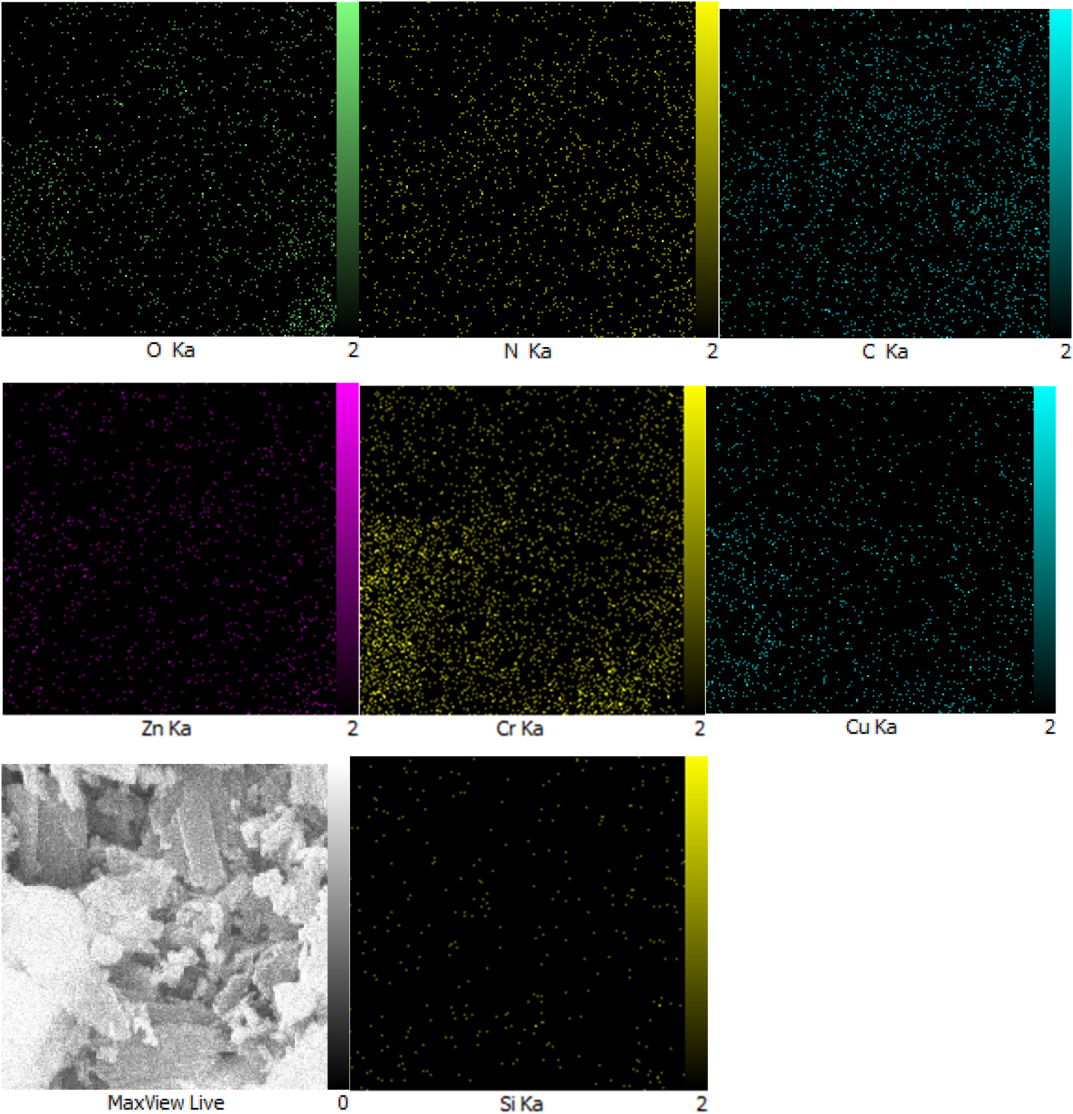
Elemental mapping analysis (MAPPING) of LDH@PTRMS@NDBD@CuI catalyst.

Moreover, another investigation involved elemental determination analysis utilizing energy dispersive spectrometer to examine the constituent elements comprising Zn/Cr-LDH@PTRMS@NDBD@CuI nanocatalyst. The outcomes of this examination validated the existence of N, C, S, O, Si, Cu, Zn, and Cr elements within the catalyst's structure (refer to Fig. 2). Furthermore, as depicted in Fig. 3, the elemental analysis of the composition reaffirms the presence of all aforementioned elements.

**Fig. 4:**
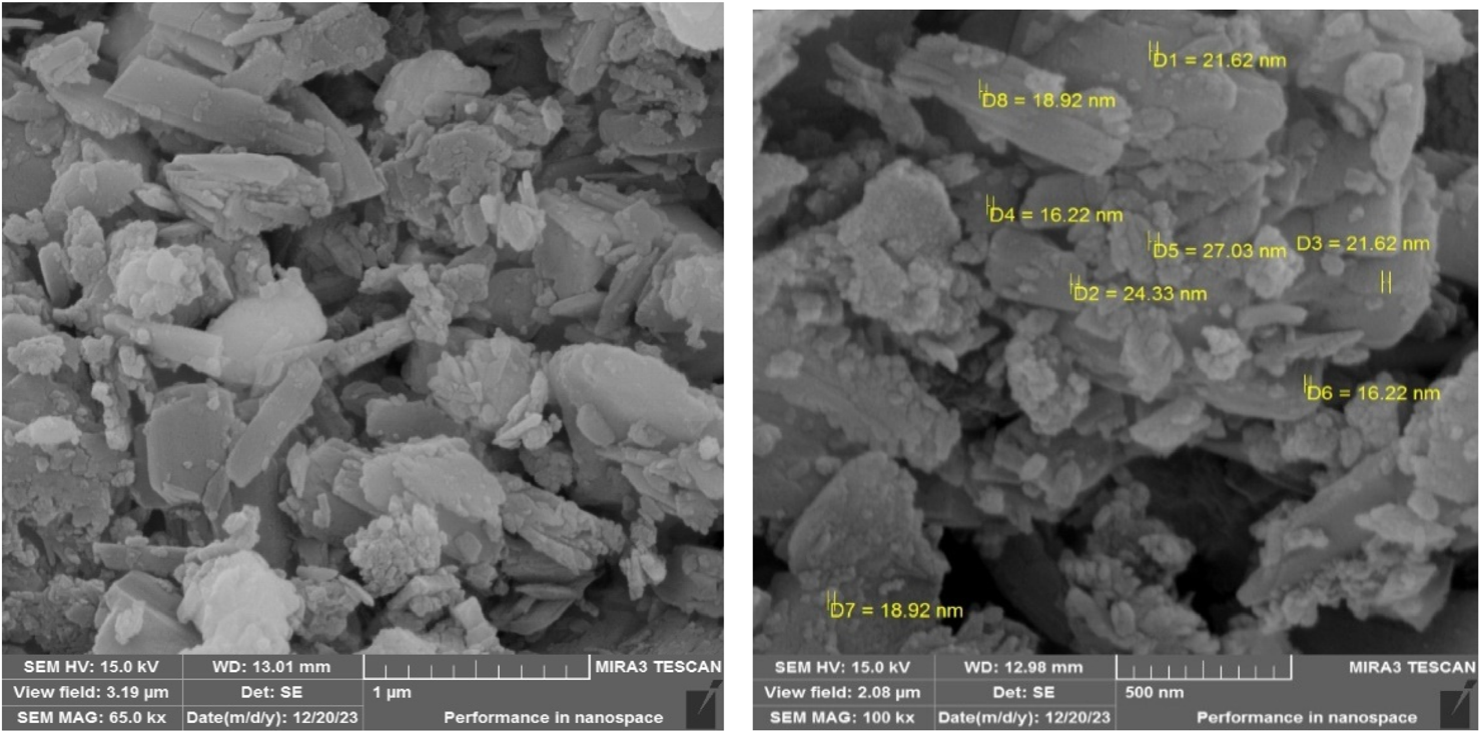
FESEM images of Zn/Cr-LDH@PTRMS@NDBD@CuI catalyst.

To explore the structure, morphology, and particle dimensions of the LDH@TRMS@NDBD@CuI nanocatalyst, images obtained from FESEM analysis are presented in Fig. 4. The image distinctly reveals the hexagonal and planar layer structure of LDH, measuring approximately 6 μm in size, indicating the preservation of its fundamental structure. Furthermore, the presence of the ligand and CuI metal results in surface roughening of the catalyst, with nanoparticles displaying a consistent and spherical shape. Notably, the nanoparticle size falls within the range of 18 to 28 nm.

**Fig. 5:**
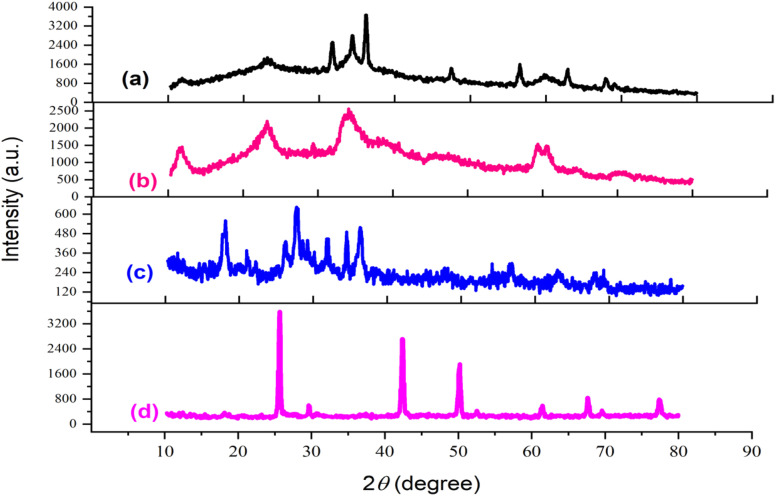
X-ray diffraction of the catalyst and its intermediates (a) double hydroxide layer, (b) LDH@PTRMS, (c) LDH@PTRMS@NDBD and (d) LDH@PTRMS@NDBD@CuI.

X-ray diffraction (XRD) analysis spanning the 2*θ* region from 10 to 80° was conducted to characterize the catalyst synthesis stages. The patterns corresponding to (a) LDH, (b) LDH@PTRMS, (c) LDH@PTRMS@NDBD, and (d) LDH@PTRMS@NDBD@CuI are depicted in Fig. 5. In part (a), the peaks at 11°, 24°, 34°, 35°, 37°, 48°, 58°, 60°, and 68° correspond respectively to the crystal planes (003), (006), (101), (012), (015), (018), (108), (110), and (113), matching the zinc/chrome-LDH structure.^48^ In part (b), related to the activation of the LDH surface with 3-chlorotrimethoxysilane, all the peaks from the previous step are observed with different intensities. Part (c) involves the placement of the ligand on the surface of the activated LDH, leading to changes in the intensity and value of the peaks from the previous step, attributed to the strong interaction with the NDBD ligand. Notably, in part (d), the peaks observed at 10°, 20°, 25°, 40°, 50°, 60°, 70°, and 98° correspond to copper,^49^ confirming the successful incorporation of copper iodide metal onto the catalyst. Estimation of the particle size using Scherer's equation reveals an approximate size of 18 nm.

**Fig. 6:**
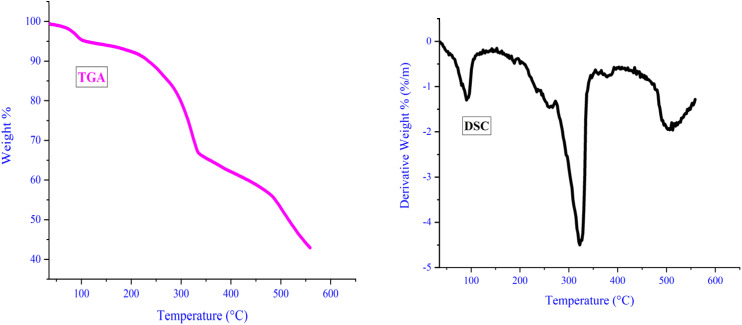
TGA and DSC thermal analyzes of Zn/Cr-LDH@PTRMS@NDBD@CuI catalyst.

In order to evaluate the catalyst's thermal stability, thermal gravimetric analysis (TGA) was carried out. As illustrated in Fig. 6, the initial weight loss, occurring around 100 °C, likely stems from the evaporation of water and solvents utilized during catalyst synthesis. Subsequent weight loss, observed between 332 and 480 °C, can be attributed to the degradation and dissociation of the ligand bound to LDH. Above 525 °C, further weight loss indicates metal removal and catalyst decomposition. Notably, all discontinuities observed in TGA analysis align with variations noted in differential scanning calorimetry (DSC) analysis. DSC analysis corroborates TGA findings and underscores the catalyst's stability up to 330 °C. The catalyst's ability to withstand such elevated temperatures is credited to the robust interaction between the ligand and LDH, further strengthened by its complexation with copper iodide. This robust interaction serves as an effective safeguard against catalyst decomposition even at lower temperatures.

### 3.2. Catalytic application of LDH@TRMS@NDBD@CuI in the preparation of tetrahydrobenzo[*b*]pyrans and 2-amino-4*H*-chromenes derivatives

Upon confirming the synthesis of the LDH@PTRMS@NDBD@CuI catalyst through the aforementioned analyses, its efficacy in catalyzing the synthesis of tetrahydrobenzo[*b*]pyrans and 2-amino-4*H*-chromenes was investigated *via* a one-pot three-component reaction. To achieve this, a model reaction involving 4-chlorobenzaldehyde (1 mmol), malononitrile (1 mmol), and dimedone (1 mmol) was employed.

In order to fine-tune the reaction parameters and assess catalyst efficiency, various parameters including temperature, catalyst quantity, solvent choice, and reaction duration were meticulously examined. Additionally, a trial run was performed without the catalyst, yielding a small output. Optimal conditions were determined to be at room temperature with 0.05 g of catalyst. Notably, the protocol's eco-friendly was demonstrated through solvent-free and room temperature reactions. The effective interaction between copper within the catalyst and the raw materials facilitated high product yields within short timeframes and without solvent usage. Decreased efficiency was observed with lower catalyst quantities, underscoring the catalyst's high effectiveness.

As indicated in Table 1, the model reaction was conducted utilizing ethanol, methanol, ethyl acetate, water, and normal hexane, resulting in a favorable yield. Notably, ethanol solvent exhibited superior yield compared to others. However, the highest efficiency was observed in solvent-free conditions, although the reaction yielded negligible results in the absence of catalyst. Furthermore, the model reaction was investigated with the inclusion of catalytic intermediates, with a summary provided in Table 2. Analysis of the table revealed that LDH, when combined with 3-chlorotrimethoxysilane on its surface, yielded 38% product within half an hour. While LDH alone exhibited consistent product yield, the introduction of the ligand onto the LDH@TRMS surface increased yield to 58%. Remarkably, the highest yield of 96% was achieved within 5 minutes with copper iodide immobilized on the LDH@TRMS@NDBD surface.

**Table 1:** Optimization of reaction conditions for the synthesis of 2-amino-4-(4-chlorophenyl)-7,7-dimethyl-5-oxo-5,6,7,8-tetrahydro-4*H*-chromen-3-carbonitrile derivative

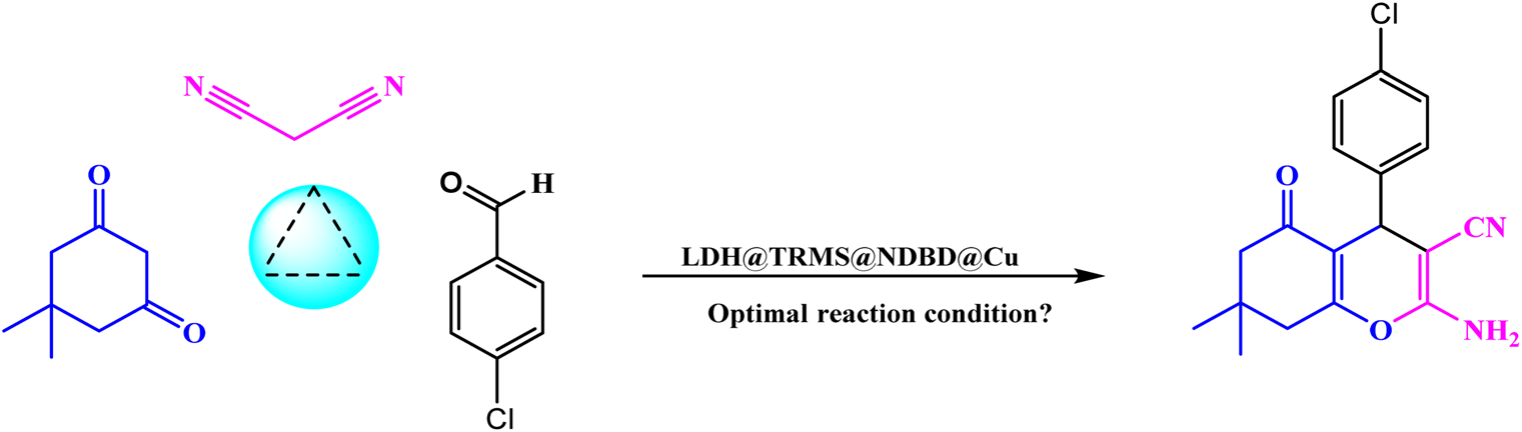					
Entry	Solvent	Load of catalyst (mg)	Temperature (°C)	Time (min)	Yield (%)
1	—	—	70	60	28
2	—	10	r.t	15	70
3	—	10	40	13	73
4	—	20	r.t	12	81
5	—	20	40	10	84
6	—	35	r.t	11	87
7	—	40	40	12	89
8	—	45	50	7	94
9	—	50	r.t	6	92
10	—	55	40	6	96
11	—	50	40	5	96
12	—	50	60	5	96
13	—	50	80	3	96
14	EtOH	45	40	7	92
15	MeOH	45	40	9	45
16	H_2_O	45	40	10	75
17	EtOAc	45	40	13	88
18	*n*-Hexane	45	40	20	72

**Table 2:** Comparison of the catalytic activity of Zn/Cr-LDH@PTRMS@NDBD@CuI and its related intermediates by performing the model reaction^a^

Entry	Catalyst	Time	Yield^b^ (%)
1	LDH	20	38
2	LDH@TRMS	20	40
3	LDH@TRMS@NDBD	20	58
4	LDH@TRMS@NDBD@CuI	5	96

a Reaction condition: malononitrile (1 mmol), dimedone (1 mmol) and 4-chlorobenzaldehyde (1 mmol).

b Isolated yields.

Table 3 details the optimization conditions for the model reaction involving 4-chlorobenzaldehyde (1 mmol), malononitrile (1 mmol), and resorcinol (1 mmol), akin to Table 1. This investigation aimed to establish optimal conditions by varying catalyst amounts, temperatures, solvents, and even conducting reactions without solvent, as outlined in Table 3. The most favorable conditions were achieved at a temperature of 40 °C, without solvent, and within a reaction duration of 10 minutes, resulting in an efficiency of 94%.

**Table 3:** Refining the reaction conditions for synthesizing the derivative 2-amino-4-(4-chlorophenyl)-7-hydroxy-4*H*-chromene-3-carbonitrile

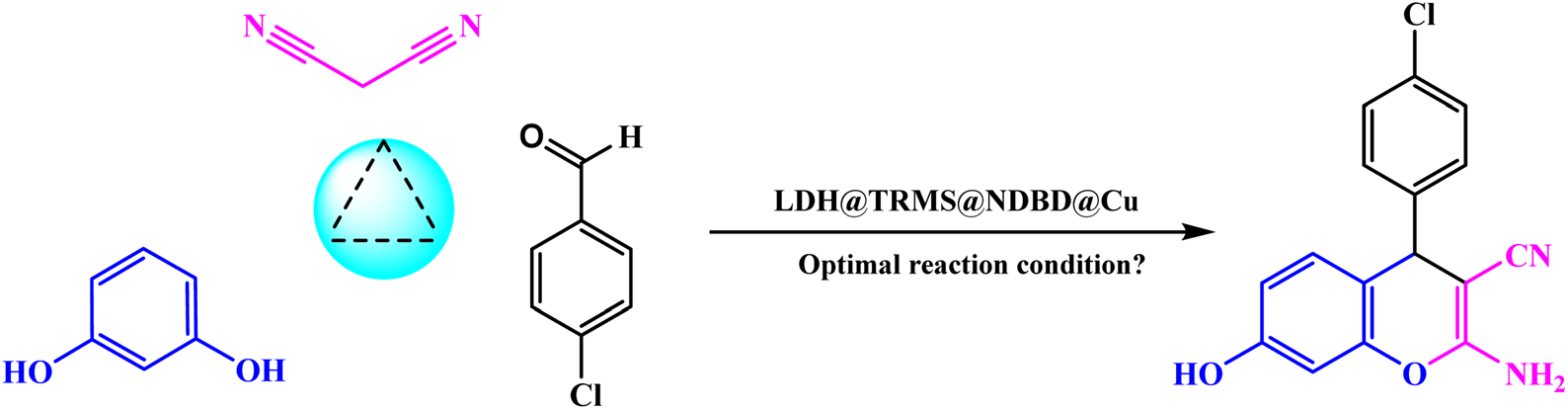					
Entry	Solvent	Load of catalyst (mg)	Temperature (°C)	Time (min)	Yield (%)
1	—	—	70	60	22
2	—	10	r.t	60	68
3	—	10	40	45	71
4	—	20	r.t	37	82
5	—	20	40	30	84
6	—	35	r.t	30	85
7	—	40	40	17	89
8	—	45	50	15	91
9	—	50	r.t	16	92
10	—	55	40	10	94
11	—	50	40	10	94
12	—	50	60	9	94
13	—	50	80	8	96
14	EtOH	45	40	7	80
15	MeOH	45	40	9	45
16	H_2_O	45	40	10	Trace
17	EtOAc	45	40	13	50
18	CH_3_CN	45	40	20	Trace

On the other hand, Table 4 presents the model reaction involving 4-chlorobenzaldehyde, malononitrile, and resorcinol in the presence of catalyst intermediates. Notably, when LDH was utilized, 25% product was obtained in 20 minutes. Incorporating 3-chlorotrimethoxysilane onto the LDH surface increased product yield to 38%, whereas introduction of the ligand onto the LDH@TRMS surface further elevated yield to 56%. Remarkably, the highest product yield of 94% was achieved within 10 minutes with copper iodide immobilized on the LDH surface. TRMS@NDBD configuration exhibited this optimal performance.

**Table 4:** Comparison of the catalytic activity of Zn/Cr-LDH@PTRMS@NDBD@CuI and its related intermediates by performing the model reaction^a^

Entry	Catalyst	Time	Yield^b^ (%)
1	LDH	20	25
2	LDH@TRMS	20	38
3	LDH@TRMS@NDBD	20	56
4	LDH@TRMS@NDBD@CuI	10	94

a Reaction condition: malononitrile (1 mmol), resorcinol (1 mmol) and 4-chlorobenzaldehyde (1 mmol).

b Isolated yields.

To evaluate the efficacy of the novel LDH@PTRMS@NDBD@CuI catalyst, the synthesis of tetrahydrobenzo[*b*]pyrans and 2-amino-4*H*-chromenes derivatives was conducted under optimal conditions. Utilizing malononitrile, dimedone or resorcinol, and a diverse array of benzaldehydes featuring various electron-donating and electron-withdrawing substituents, the reactions were performed swiftly, yielding high product yields (Table 5). Benzaldehydes featuring electron-withdrawing groups positioned *ortho* and *para* displayed increased product yields and reduced reaction times. This enhancement can be ascribed to the heightened reactivity of carbonyl benzaldehydes bearing electron-withdrawing groups, which readily react with malononitrile in the catalyst's presence.

**Table 5:** Synthesis of tetrahydrobenzo[*b*]pyrans and 2-amino-4*H*-chromenes derivatives in the vicinity of Zn/Cr-LDH@PTRMS@NDBD@CuI catalyst ^40,46,50–53^

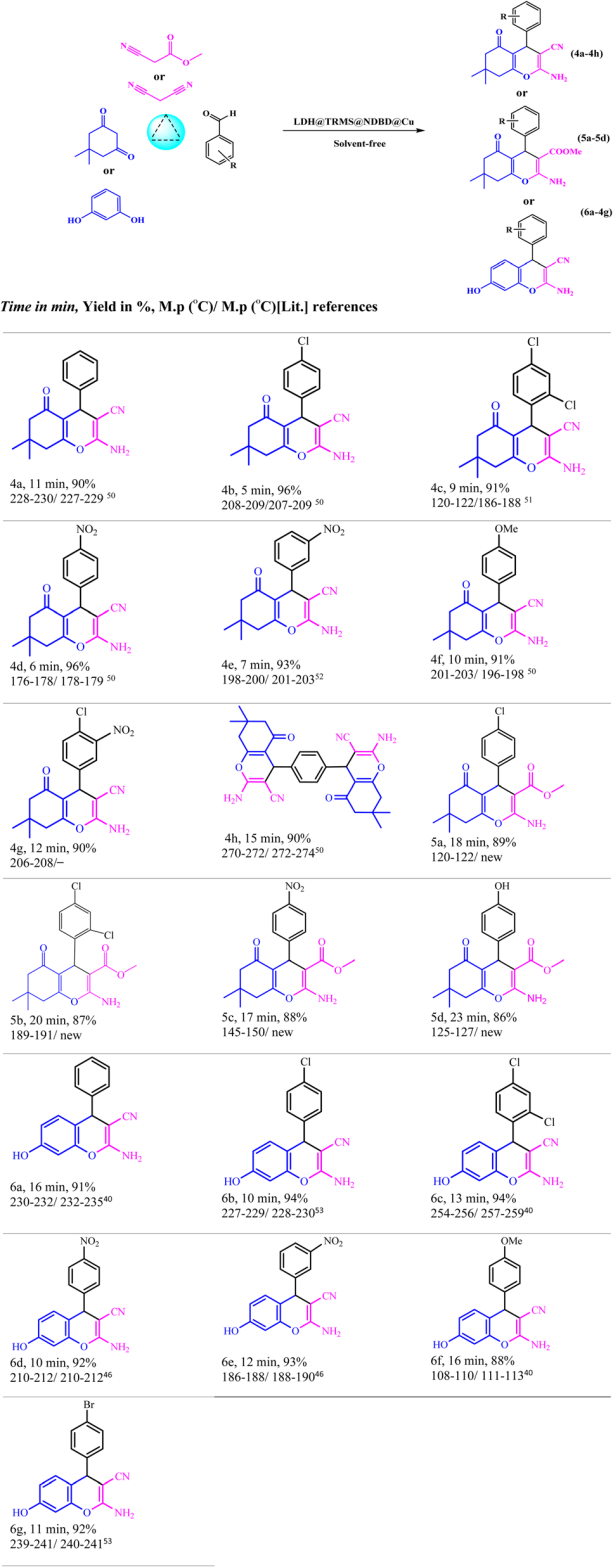

**Fig. 7:**
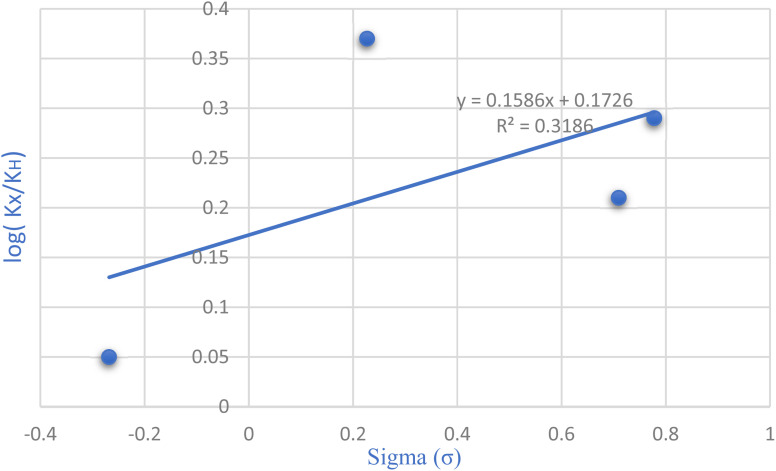
Exploring substitution groups in the synthesis of tetrahydrobenzo[*b*]pyrans *via* the Hammett plot.

The Hammett equation, a fundamental linear free energy equation, delineates the correlation between reaction energetics and the impact of substituents. Changes in the reaction's free energy consequently influence the activation energy of the transition state. Plotting the ratio of rate constants log(*k*_X_/*k*_H_) against the substituent constant (*σ*) for various substituent groups located meta and para to the reaction center, as depicted in Fig. 7 of the Hammett diagram, reveals discernible trends. Notably, the positive slope evident in the Hammett reaction diagram signifies that electron-withdrawing groups augment the reaction rate. However, they lack a substantial impact on the reaction yield.

**Scheme 2:**
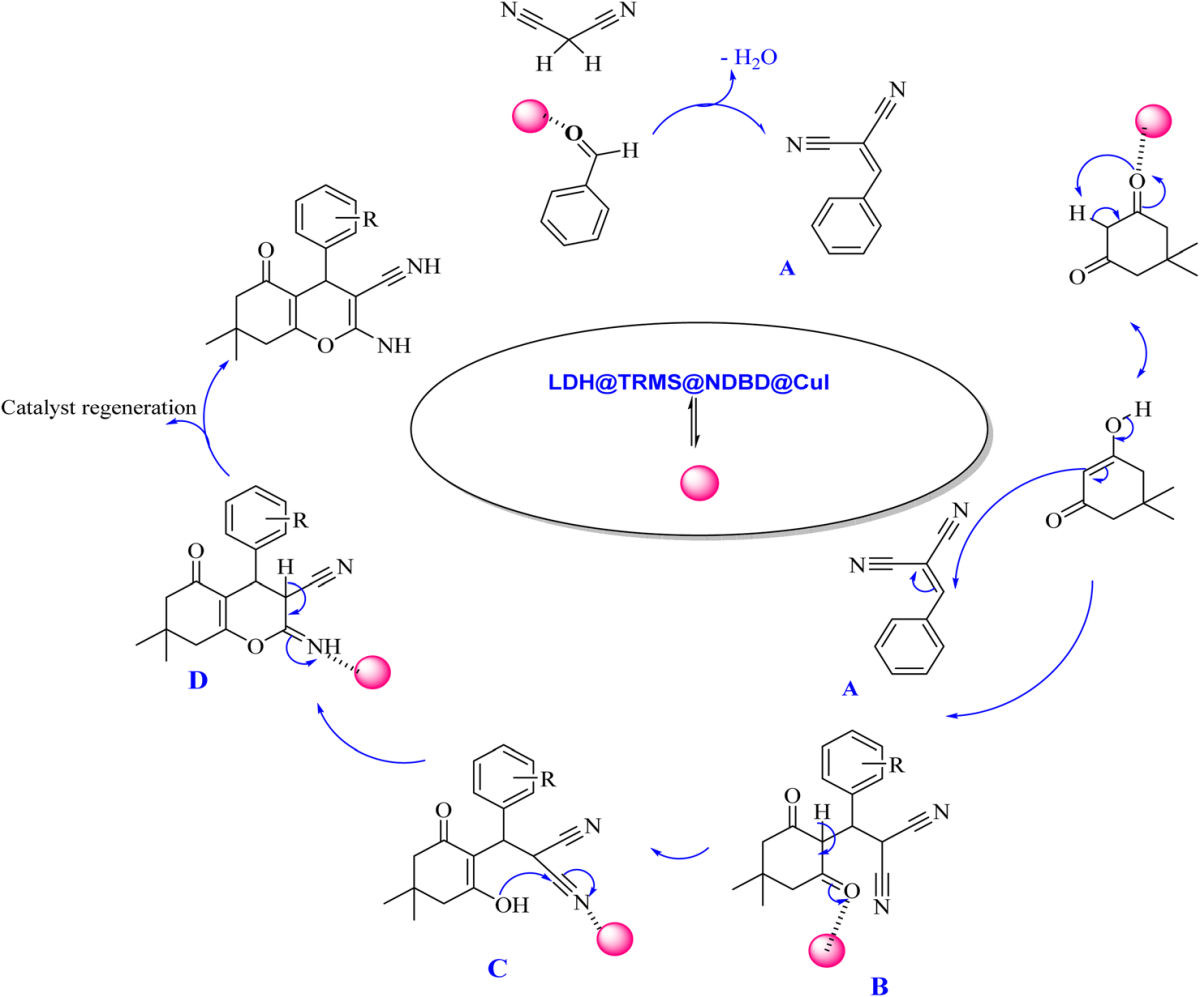
Proposed mechanism for the synthesis of tetrahydrobenzo[*b*]pyran derivatives in the vicinity of Zn/Cr-LDH@PTRMS@NDBD@CuI catalyst.

**Scheme 3:**
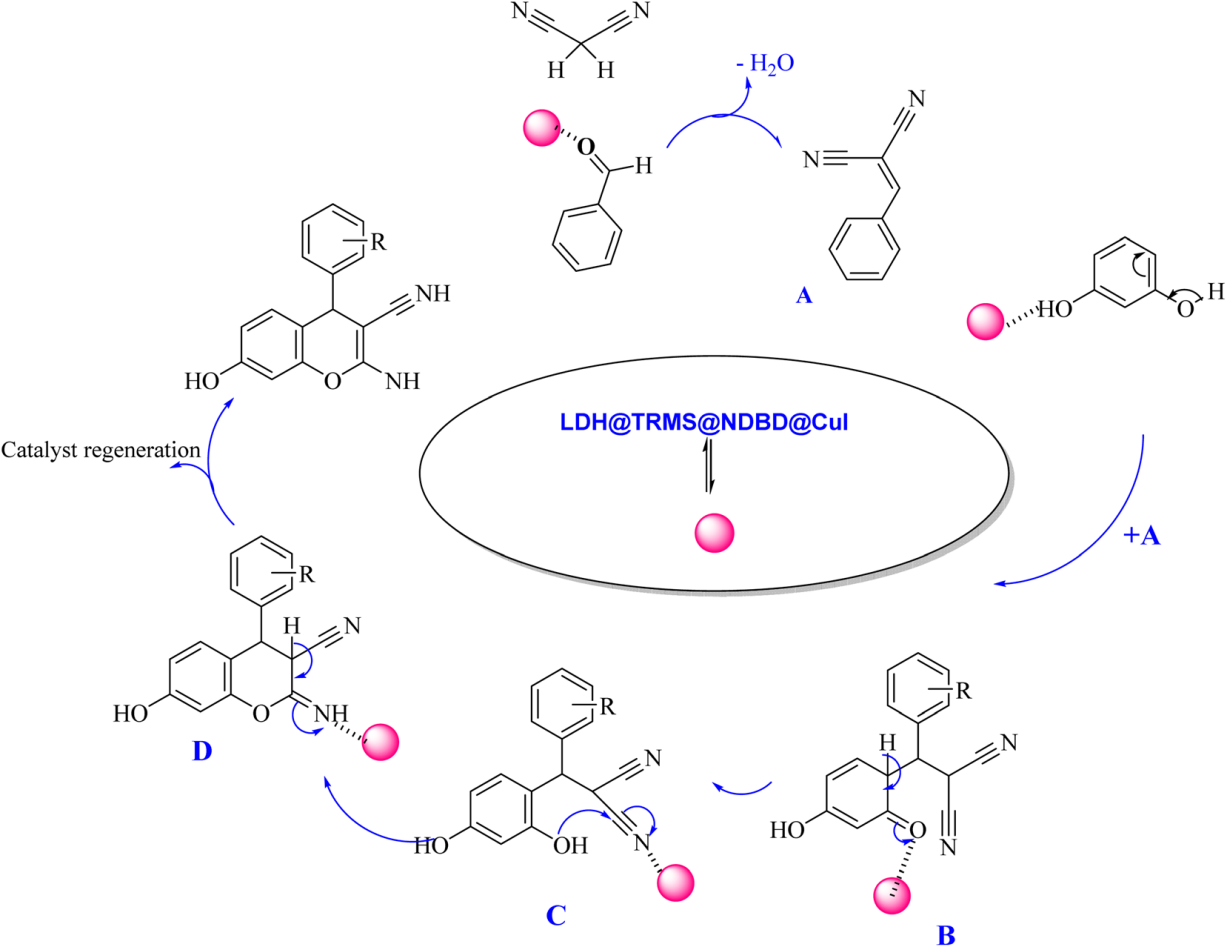
Proposed mechanism of synthesis of 2-amino-4*H*-chromenes derivatives in the vicinity of Zn/Cr-LDH@PTRMS@NDBD@CuI catalyst.

The proposed mechanism elucidating the synthesis of tetrahydrobenzo[*b*]pyrans and 2-amino-4*H*-chromenes derivatives is depicted in Schemes 2 and 3, respectively, detailing the catalytic pathway. As delineated in Scheme 2, the process initiates with the activation of carbonyl benzaldehyde by the nanocatalyst, rendering it susceptible to nucleophilic attack by activated malononitrile, forming intermediate A. Subsequently, intermediate A undergoes a reaction with the enolic form of dimedone, yielding intermediate B. With the aid of the catalyst, intermediate B undergoes proton tautomerism to generate intermediate C, followed by intramolecular attack by intermediate C to yield intermediate D. Tautomerization of intermediate D culminates in product formation in the vicinity of the catalyst. Furthermore, the catalyst exhibits recyclability, facilitating its reuse in subsequent cycles.

The proposed mechanism delineating the synthesis of 2-amino-4-*H*-chromenes derivatives is illustrated in Scheme 3. According to this scheme, it becomes evident that intermediate A, activated by the nanocatalyst, primes carbonyl benzaldehyde for nucleophilic attack by malononitrile. This activated state facilitates the reaction between intermediate A and activated resorcinol, leading to the generation of intermediate B. With the catalyst's presence, intermediate B undergoes proton tautomerism, transitioning to intermediate C. Subsequently, intermediate C executes an intramolecular attack, yielding intermediate D. Subsequent tautomerization of intermediate D within the catalyst's vicinity leads to product synthesis, while the catalyst is effectively recycled, reinstated for the next reaction cycle.

**Fig. 8:**
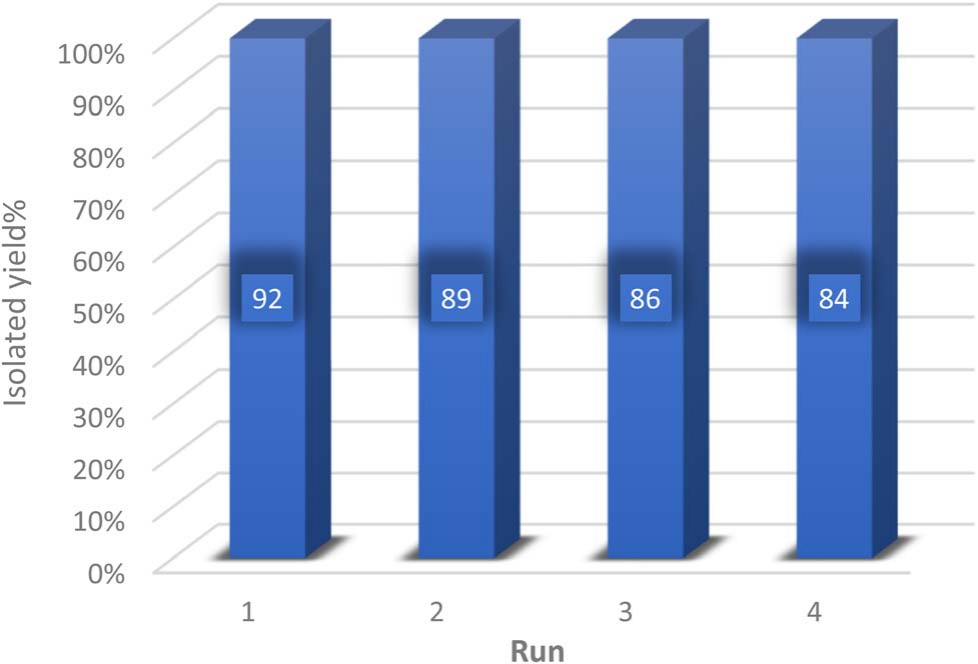
Ability to recycle and reuse Zn/Cr-LDH@PTRMS@NDBD@CuI catalyst.

To explore the feasibility of recycling and reutilizing the catalyst, a model reaction involving dimedone (1 mmol), 4-chlorobenzaldehyde (1 mmol), and malononitrile (1 mmol) was conducted in the presence of the catalyst. Following the reaction's completion, either ethyl acetate or ethanol was introduced into the reaction mixture, causing the synthesized product to dissolve in the solvent while the insoluble catalyst was separated *via* centrifugation. Subsequently, the separated catalyst was dried in an oven at 60 °C and utilized in the subsequent reaction. As depicted in Fig. 8, the catalyst was recycled four times without any significant decrease in its efficiency.

In order to demonstrate the effectiveness of the newly developed catalyst, a comparative study was undertaken, evaluating it against alternative catalysts in the synthesis of tetrahydrobenzo[*b*]pyrans and 2-amino-4*H*-chromenes through a three-component reaction. The detailed findings are presented in Tables 6 and 7. The results depicted in these tables underscore the considerable enhancement afforded by the current catalyst in optimizing reaction conditions. Remarkably, with the LDH@PTRMS@NDBD@CuI catalyst, both reaction time and temperature decreased, while product yield increased, underscoring the heightened efficiency of the current catalyst.

**Table 6:** Assessing the effectiveness of the Zn/Cr-LDH@PTRMS@NDBD@CuI catalyst in producing tetrahydrobenzo[*b*]pyran derivatives relative to other catalystsis

Entry	Reaction conditions	Time (min)	Yield (%)	Lit.
1	MCM-41@Schiff base-Co(OAc)_2_, H_2_O, 50 °C	180	94	54
2	rGO@Fe_3_O_4_-ZrCp_2_Cl_2_, PEG-400, 100 °C	120	80	55
3	Fe_3_O_4_@GO-*N*-(pyridin-4-amine), H_2_O, reflux	30	92	56
4	NH_4_Al(SO_4_)_2_·12H_2_O (Alum)/EtOH/80 °C	130	90	57
5	NiFe_2_O_4_@SiO_2_-H_3_PW_12_O_40_, EtOH, reflux	15	89	58
6	PPI/H_2_O/reflux	15	90	59
7	Fe_3_O_4_@SiO_2_/DABCO, H_2_O, reflux	25	90	60
8	Fe_3_O_4_@MCM-41@Zr-piperazine-MNPs, EtOH/H_2_O (3 : 7)/reflux	30	85	50
9	LDH@PTRMS@NDBD@CuI, solvent free, 40 °C	5	96	This work

**Table 7:** Assessing the effectiveness of the Zn/Cr-LDH@PTRMS@NDBD@CuI catalyst in producing 2-amino-4*H*-chromenes derivatives relative to other catalysts

Entry	Reaction conditions	Time (min)	Yield (%)	Lit.
1	PdRu@GO, H_2_O : EtOH (2 : 1), 80 °C	12	93	61
2	Fe(HSO_4_)_3_, refluxed in MeCN_2_	240	83	62
3	Amino-appended β-cyclodextrin (a3), H_2_O, r.t	180	94	63
4	Tungstic acid functionalized mesoporous SBA-15 (TAFMC-1), H_2_O, reflux	720	80	38
5	TPOP-2 (40 mg), solvent-free, 80 °C	240	88	64
6	Sodium carbonate, EtOH/H_2_O, 25 °C	84	120	45
7	POPINO, H_2_O, reflux	15	92	43
8	Mg/Al: 5.0 hydrotalcite (HT), H_2_O, 80 °C	300	90	46
9	LDH@PTRMS@NDBD@CuI, solvent free, 40 °C	10	94	This work

## 4. Conclusion

In brief, a novel approach was devised for fabricating the LDH@PTRMS@NDBD@CuI nanocatalyst, integrating copper iodide nanoparticles onto a nanocomposite derived from a modified layered double hydroxide with *N*_1_,*N*_4_-bis(4,6-diamino-1,3,5-triazin-2-yl)benzene-1,4-disulfonamide (NDBD). Validation of this catalyst was meticulously conducted using various instrumental techniques, demonstrating its remarkable efficacy and recyclability when deployed in a one-pot three-component reaction for synthesizing tetrahydrobenzo[*b*]pyrans and 2-amino-4*H*-chromenes compounds under soft conditions. Notably, this method champions green chemistry principles by eliminating toxic organic solvents. Some of its main benefits include using easily accessible raw materials, a simplified single-step reaction process, reduced reaction time, simple product purification, eco-friendly, and increase product yield. Furthermore, the catalyst exhibits exceptional reusability, maintaining significant efficiency over up to four cycles with ease of extraction from the reaction mixture. This catalyst holds promise for synthesizing new heterocycles, highlighting its prospective utility in forthcoming endeavors.

## Data availability

The data supporting this article have been included as part of the ESI.†

## Author contributions

R. G. H. and S. M. conceived the experiments and provided experimental instructions, S. M. performed the experiments, analyzed the results and wrote the manuscript. The authors reviewed and revised the manuscript.

## Conflicts of interest

The authors declare no competing interests.

## Supplementary Material

RA-014-D4RA04239E-s001
