# Supplementary material for: A facile one-pot synthesis of tetrahydrobenzo[b]pyrans and 2-amino-4H-chromenes under green conditions
Source: RSC Adv. 2024 Jul 16;14(30):21608–22. doi: 10.1039/d4ra04239e (PMC11249666; doi:10.1039/d4ra04239e)
Supplement: RA-014-D4RA04239E-s001 [file RA-014-D4RA04239E-s001.pdf]

Electronic Supplementary Material (ESI) for RSC Advances.
This journal is © The Royal Society of Chemistry 2024

Electronic Supplementary Information (ESI)

A facile one-pot synthesis of tetrahydrobenzo[*b*]pyrans and 2-amino-4*H*-chromenes under green conditions

Sarieh Momeni, Ramin Ghorbani-Vaghei*

Department of Organic Chemistry, Faculty of Chemistry and Petroleum Sciences, Bu-Ali Sina University, 6517838683, Hamadan, *Iran*

**Corresponding author; E-mail: rgvaghei@yahoo.com & ghorbani@basu.ac.ir*

Spectral Data:

S1. 2-amino-4-(4-chlorophenyl)-7,7-dimethyl-5-oxo-5,6,7,8-tetrahydro-4H-chromene-3-carbonitrile

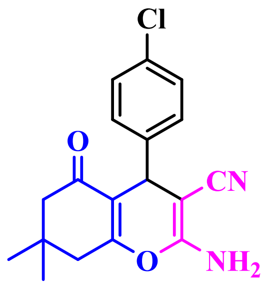

Melting point=190-192 °C,

FT-IR (KBr, ν cm^{-1}): 3460, 3381, 3325, 3185, 2959, 2189, 1675, 1655, 1636, 1491, 1366, 1215, 1033, 855, 771, 563; ^{1}H NMR (250 MHz, DMSO-d_6) δ 7.32 (d, J = 7.9 Hz, 2H), 7.15 (d, J = 7.6 Hz, 2H), 7.01 (d, J = 4.3 Hz, 2H), 4.17 (s, 1H), 2.49 (s, 2H), 2.38 – 1.94 (m, 2H), 1.00 (d, J = 4.2 Hz, 3H), 0.92 (d, J = 4.0 Hz, 3H); ^{13}C NMR (63 MHz, DMSO-d_6) δ 196.10, 163.05, 158.96, 144.17, 131.55, 129.55, 128.72, 119.96, 112.79, 58.26, 50.40, 35.56, 32.22, 28.73, 27.31.

2-amino-7,7-dimethyl-4-(4-nitrophenyl)-5-oxo-5,6,7,8-tetrahydro-4H-chromene-3-carbonitrile

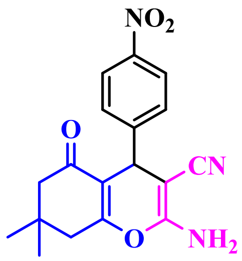

FT-IR (KBr, ν cm^{-1}): 3411, 3330, 3256, 3213, 2959, 2192, 1682, 1659, 1594, 1518, 1366, 1346, 1253, 1214, 1039, 829, 700, 562; ^{1}H NMR (250 MHz, DMSO-d_6) δ 8.15 (d, J = 8.2 Hz, 2H), 7.42 (d, J = 8.2 Hz, 2H), 7.16 (s, 2H), 4.34 (s, 1H), 2.51 (s, 2H), 2.33 – 2.01 (m, 2H), 1.02 (s, 3H), 0.93 (s, 3H). ^{13}C NMR (63 MHz, DMSO-d_6) δ 196.16, 163.57, 159.06, 152.74, 146.73, 129.07, 124.12, 119.77, 112.18, 57.43, 50.31, 36.09, 32.27, 28.70, 27.38.

2-amino-4-(4-chloro-3-nitrophenyl)-7,7-dimethyl-5-oxo-5,6,7,8-tetrahydro-4H-chromene-3-carbonitrile

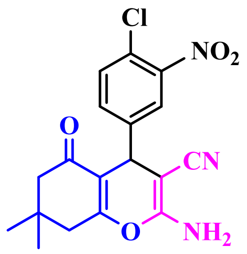

^{1}H NMR (250 MHz, DMSO-d_6) δ 7.83 (s, 1H), 7.67 (s, 1H), 7.50 (s, 1H), 7.17 (s, 2H), 4.34 (s, 1H), 2.53 (s, 2H), 2.15 (q, J = 17.5, 16.0 Hz, 2H), 0.99 (s, 3H), 0.93 (s, 3H). ^{13}C NMR (63 MHz, DMSO-d_6) δ 196.29, 163.75, 159.04, 148.00, 146.43, 133.24, 131.98, 124.53, 123.36, 119.73, 111.76, 57.23, 50.32, 35.45, 32.25, 28.57, 27.49.

2-amino-4-(2,4-dichlorophenyl)-7,7-dimethyl-5-oxo-5,6,7,8-tetrahydro-4H-chromene-3-carbonitrile

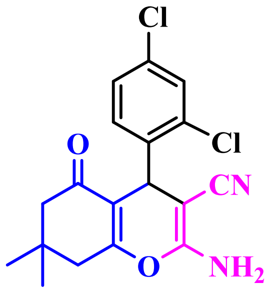

FT-IR (KBr, ν cm^{-1}): 3460, 3356, 3334, 3163, 2961, 2193, 1665, 1660, 1650, 1474, 1366, 1216, 1044, 855, 560; ^{1}H NMR (250 MHz, DMSO-d_6) δ 7.48 (s, 1H), 7.31 (s, 1H), 7.18 (s, 1H), 7.05 (s, 2H), 4.64 (s, 1H), 2.52 (s, 1H), 2.13 (q, J = 16.4, 15.4 Hz, 2H), 1.00 (s, 3H), 0.94 (s, 3H). ^{13}C NMR (63 MHz, DMSO-d_6) δ 196.04, 163.75, 159.15, 141.15, 133.46, 132.21, 131.86, 129.18, 128.07, 119.53, 111.81, 56.72, 50.34, 32.18, 28.74, 27.37.

2-amino-4-(4-methoxyphenyl)-7,7-dimethyl-5-oxo-5,6,7,8-tetrahydro-4H-chromene-3-

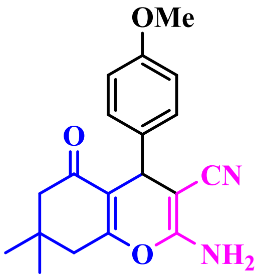

carbonitrile

^{1}H NMR (250 MHz, DMSO-d_6) δ 7.02 (d, J = 8.1 Hz, 2H), 6.93 (s, 2H), 6.81 (d, J = 8.2 Hz, 2H), 4.09 (s, 1H), 3.68 (s, 3H), 2.52 (s, 2H), 2.39 – 1.84 (m, 2H), 1.00 (s, 3H), 0.92 (s, 3H). ^{13}C NMR (63 MHz, DMSO-d_6) δ 196.10, 162.57, 158.89, 158.36, 137.28, 128.65, 120.25, 114.12, 113.43, 58.98, 55.42, 50.45, 35.17, 32.21, 28.83, 27.21.

4,4'-(1,4-phenylene)bis(2-amino-7,7-dimethyl-5-oxo-5,6,7,8-tetrahydro-4H-chromene-3-carbonitrile)

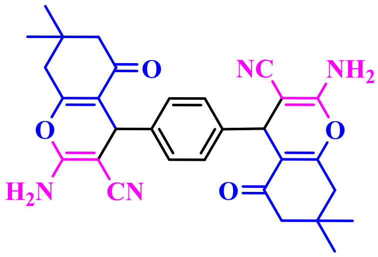

^{1}H NMR (250 MHz, DMSO-d_6) δ 7.90 (s, 3H), 7.82 (d, J = 8.6 Hz, 1H), 7.74 (d, J = 9.0 Hz, 1H), 7.60 (d, J = 9.6 Hz, 1H), 7.45 (d, J = 8.6 Hz, 1H), 7.37 – 6.87 (m, 1H), 4.87 (s, 2H), 2.67 (s, 4H), 2.32 – 2.01 (m, 4H), 1.05 (s, 6H), 0.97 (s, 6H).

methyl 2-amino-4-(4-chlorophenyl)-7,7-dimethyl-5-oxo-5,6,7,8-tetrahydro-4H-chromene-3-carboxylate

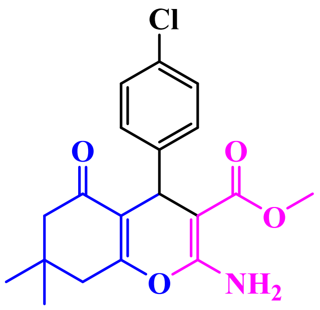

Melting point=190-192 °C,

FT-IR (KBr, ν cm^{-1}): 3429, 2961, 2879, 1729, 1594, 1491, 1373, 1276, 1206, 1091, 837, 494; ^{1}H NMR (250 MHz, DMSO-d_6) δ 1H NMR (250 MHz, DMSO-d_6) δ 7.58 (s, 2H), 7.24 (d, J = 8.4 Hz, 2H), 7.12 (d, J = 8.7 Hz, 2H), 4.46 (s, 1H), 3.47 (s, 3H), 2.20 (s, 2H), 2.07-1.97 (m, 2H), 1.04 (s, 6H).; ^{13}C NMR (63 MHz, DMSO-d_6) δ 13C NMR (63 MHz, DMSO-d_6) δ 196.43, 168.64, 162.88, 159.66, 154.25, 145.74, 132.87, 130.79, 129.93, 129.81, 128.23, 115.59, 77.63, 50.99, 50.34, 33.27, 32.29, 29.01, 26.86.

2-amino-4-(4-hydroxyphenyl)-7,7-dimethyl-5-oxo-5,6,7,8-tetrahydro-4H-chromene-3-carboxylate

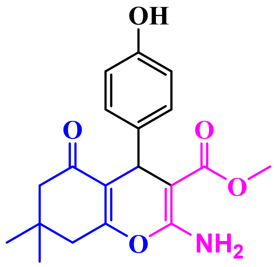

^{1}H NMR (250 MHz, DMSO-d_6) δ 9.16 (s, 1H), 6.90 (s, 3H), 6.66 (s, 1H), 6.52 (d, J = 13.8 Hz, 2H), 4.67 (s, 1H), 3.35 (s, 3H), 2.29 (s, 2H), 2.09 (s, 2H), 0.84 (s, 7H). ^{13}C NMR (63 MHz, DMSO-d_6) δ 194.84, 155.43, 149.32, 138.36, 134.46, 128.87, 114.77, 112.34, 78.24, 50.75, 50.48, 32.55, 32.11, 29.55, 26.89, 14.48.

2-amino-4-(2,4-dichlorophenyl)-7,7-dimethyl-5-oxo-5,6,7,8-tetrahydro-4H-chromene-3-carboxylate

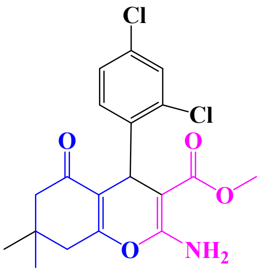

^{1}H NMR (250 MHz, DMSO-d_6) δ 8.01 (d, J = 8.6 Hz, 1H), 7.70 (d, J = 9.1 Hz, 2H), 7.62 – 7.42 (m, 2H), 7.30 – 6.91 (m, 1H), 4.28 (s, 1H), 3.87 (s, 3H), 2.29 (s, 2H), 2.12 – 1.97 (m, 2H), 1.00 (d, J = 6.9 Hz, 6H). ^{13}C NMR (63 MHz, DMSO-d_6) δ 196.07, 168.71, 159.64, 150.01, 142.64, 133.21, 131.33, 130.37, 128.96, 128.71, 127.18, 114.10, 76.34, 50.77, 50.39, 32.61, 32.20, 29.08, 26.85, 26.68.

2-amino-4-(4-chlorophenyl)-7-hydroxy-4H-chromene-3-carbonitrile

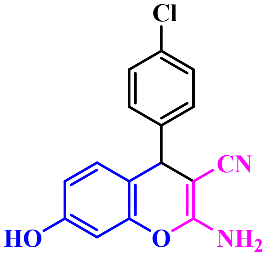

^{1}H NMR (250 MHz, DMSO-d_6) δ 9.70 (s, 1H), 7.34 (d, J = 7.9 Hz, 2H), 7.16 (d, J = 7.6 Hz, 2H), 6.89 (s, 2H), 6.83 – 6.67 (m, 1H), 6.47 (d, J = 8.0 Hz, 1H), 6.38 (s, 1H), 4.64 (s, 1H).

2-amino-7-hydroxy-4-(3-nitrophenyl)-4*H*-chromene-3-carbonitrile

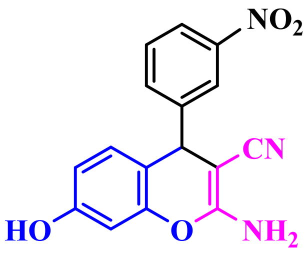

Melting point=190-192 °C,

FT-IR (KBr, ν cm^{-1}): 3440, 3335, 3217, 3078, 2194, 1645, 1586, 1530, 1407, 1351, 1157, 851, 685; ^{1}H NMR (250 MHz, DMSO-d_6) δ 1H NMR (250 MHz,) δ 9.78 (s, 1H), 8.27 – 7.88 (m, 2H), 7.63 (s, 2H), 7.03 (s, 2H), 6.83 (d, J = 8.4 Hz, 1H), 6.62 – 6.34 (m, 2H), 4.89 (s, 1H); ^{13}C NMR (63 MHz, DMSO-d_6) δ 179.41, 145.95, 137.55, 135.60, 131.51, 130.77, 130.02, 129.12, 128.89, 128.66, 128.23, 127.52, 126.97, 125.63.

2-amino-4-(2,4-dichlorophenyl)-7-hydroxy-4H-chromene-3-carbonitrile

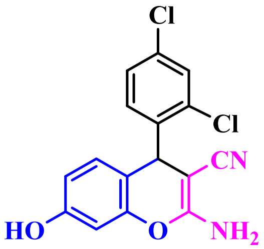

^{1}H NMR (250 MHz, DMSO-d_6) δ 9.73 (s, 1H), 7.65 (s, 2H), 7.53 (s, 1H), 7.27 (d, *J* = 44.0 Hz, 1H), 6.94 (s, 2H), 6.67 (s, 1H), 6.41 (d, *J* = 19.9 Hz, 1H), 5.09 (s, 1H).

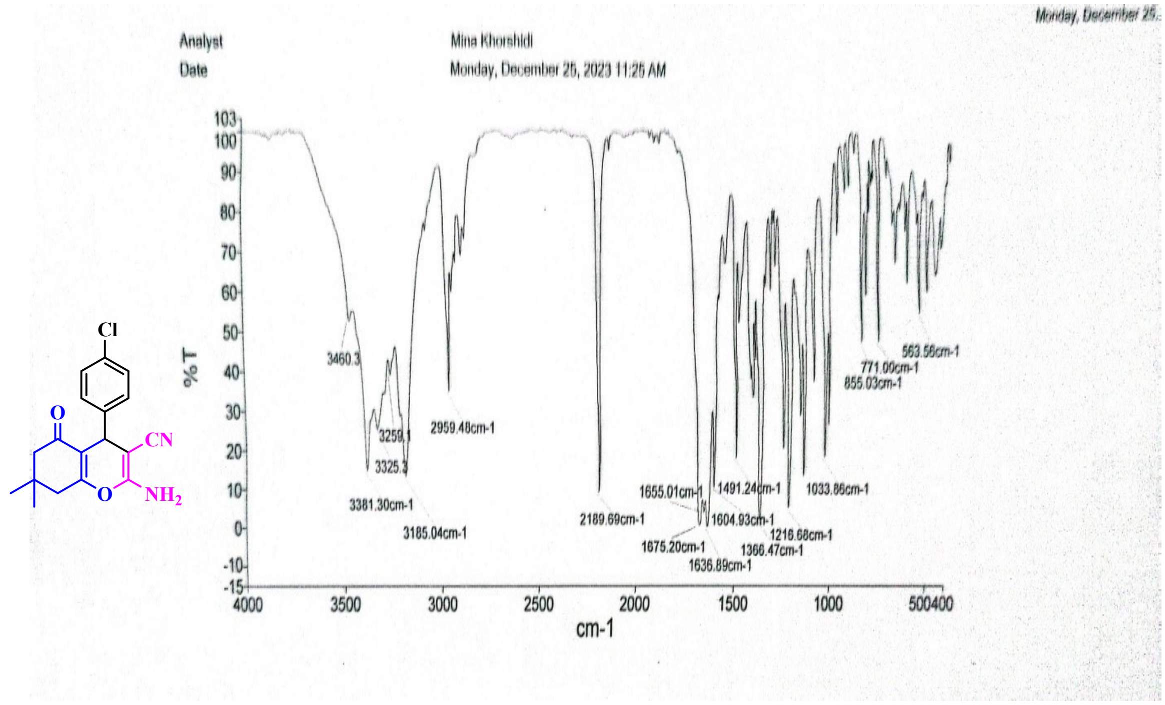

Figure 1. Spectrum FTIR of 2-amino-4-(4-chlorophenyl)-7,7-dimethyl-5-oxo-5,6,7,8-tetrahydro-4H-chromene-3-carbonitrile

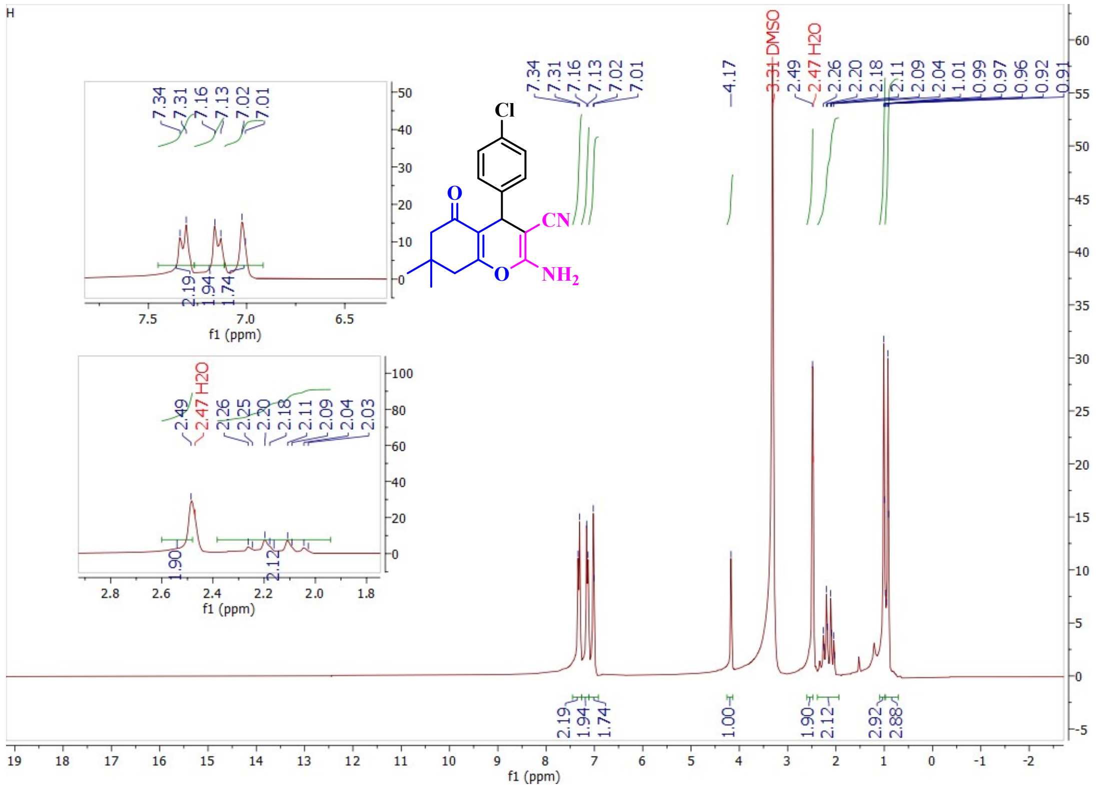

Figure 2. Spectrum ^{1}H NMR (250 MHz) of 2-amino-4-(4-chlorophenyl)-7,7-dimethyl-5-oxo-5,6,7,8-tetrahydro-4H-chromene-3-carbonitrile in DMSO solvent

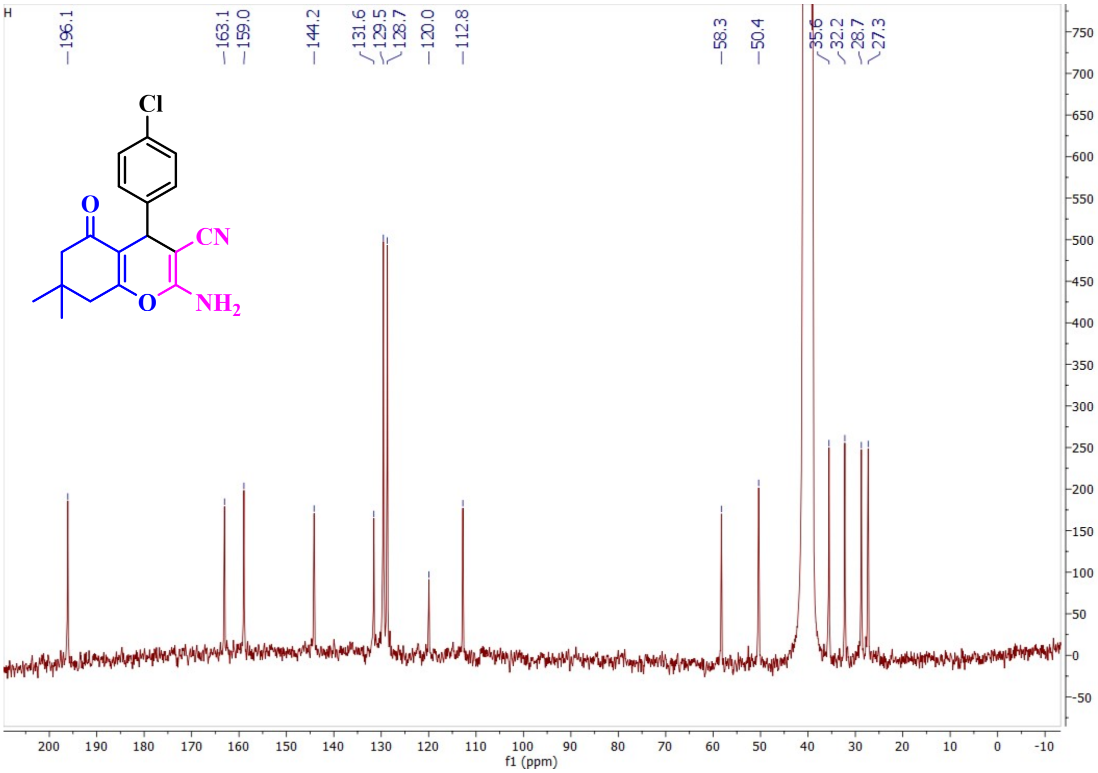

Figure 3. Spectrum ^{13}CNMR (63 MHz) of 2-amino-4-(4-chlorophenyl)-7,7-dimethyl-5-oxo-5,6,7,8-tetrahydro-4H-chromene-3-carbonitrile in DMSO solvent

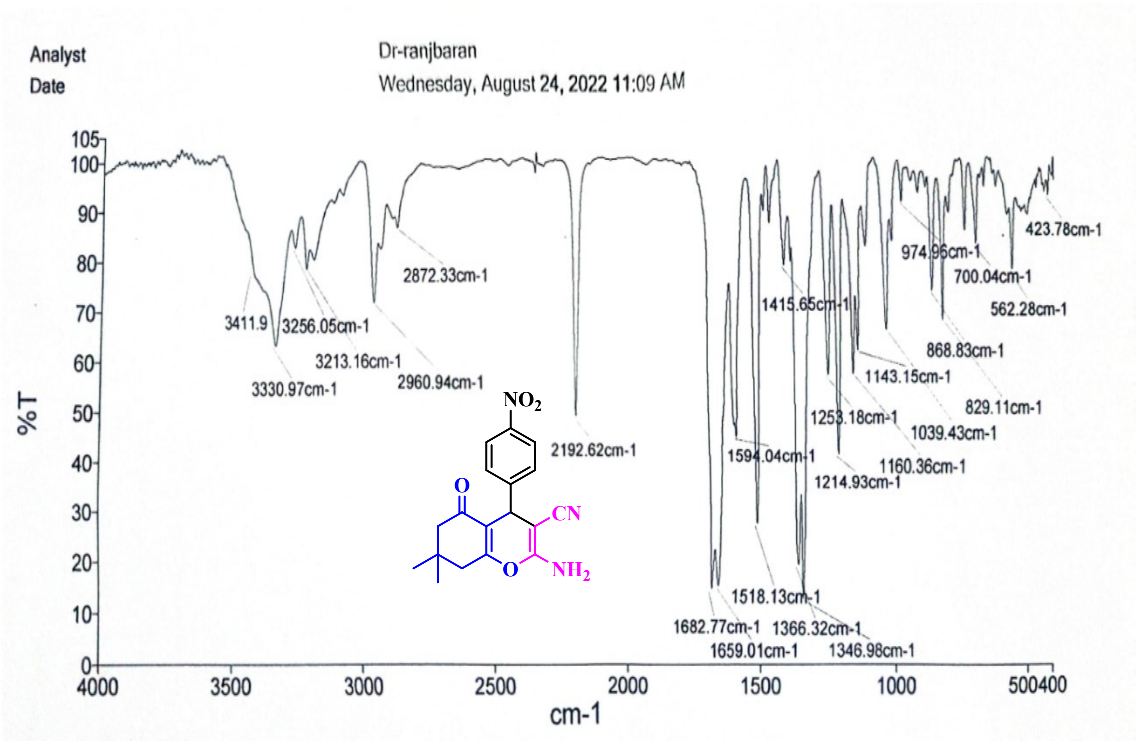

Figure 4. Spectrum FTIR of 2-amino-7,7-dimethyl-4-(4-nitrophenyl)-5-oxo-5,6,7,8-tetrahydro-4H-chromene-3-carbonitrile

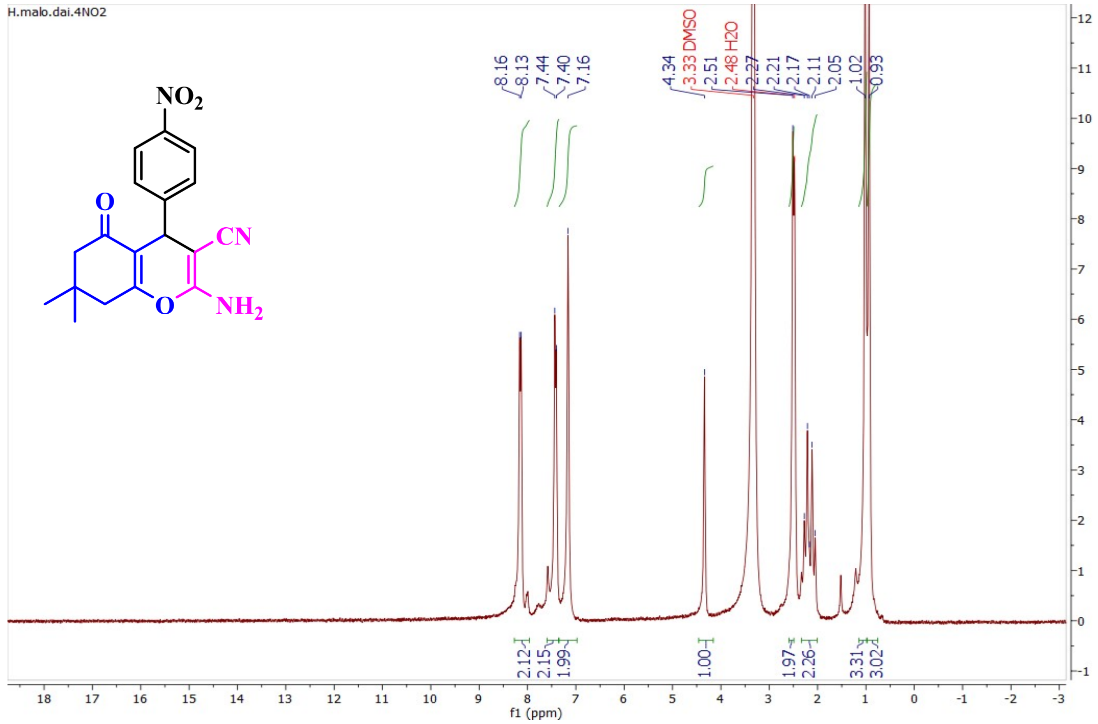

Figure 5. Spectrum ^{1}H NMR (250 MHz) of 2-amino-7,7-dimethyl-4-(4-nitrophenyl)-5-oxo-5,6,7,8-tetrahydro-4H-chromene-3-carbonitrile in DMSO solvent

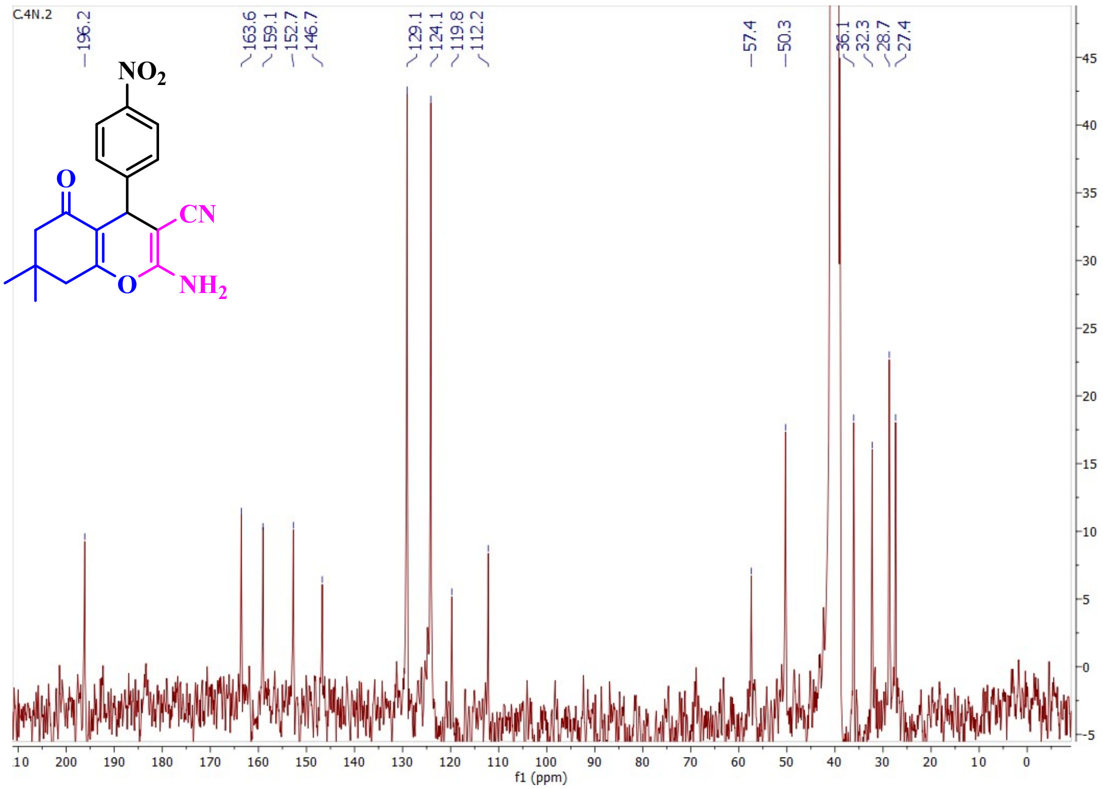

Figure 6. Spectrum ^{13}CNMR (63 MHz) of 2-amino-7,7-dimethyl-4-(4-nitrophenyl)-5-oxo-5,6,7,8-tetrahydro-4H-chromene-3-carbonitrile in DMSO solvent

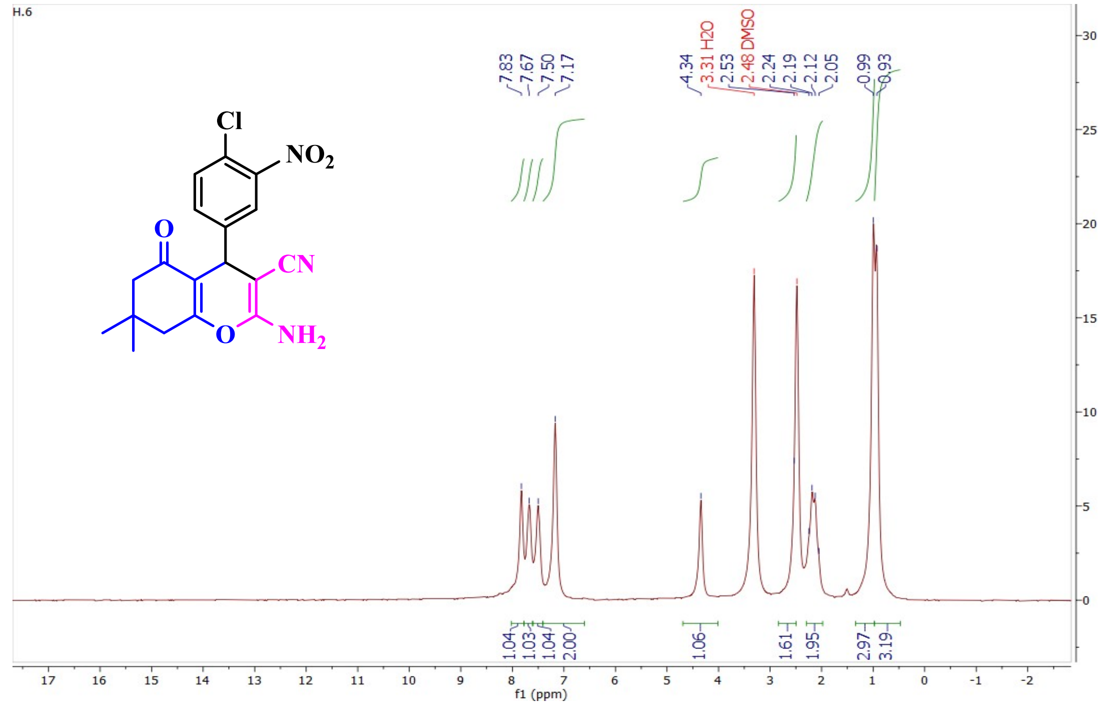

Figure 7. Spectrum ^{1}H NMR (250 MHz) of 2-amino-4-(4-chloro-3-nitrophenyl)-7,7-dimethyl-5-oxo-5,6,7,8-tetrahydro-4H-chromene-3-carbonitrile in DMSO solvent

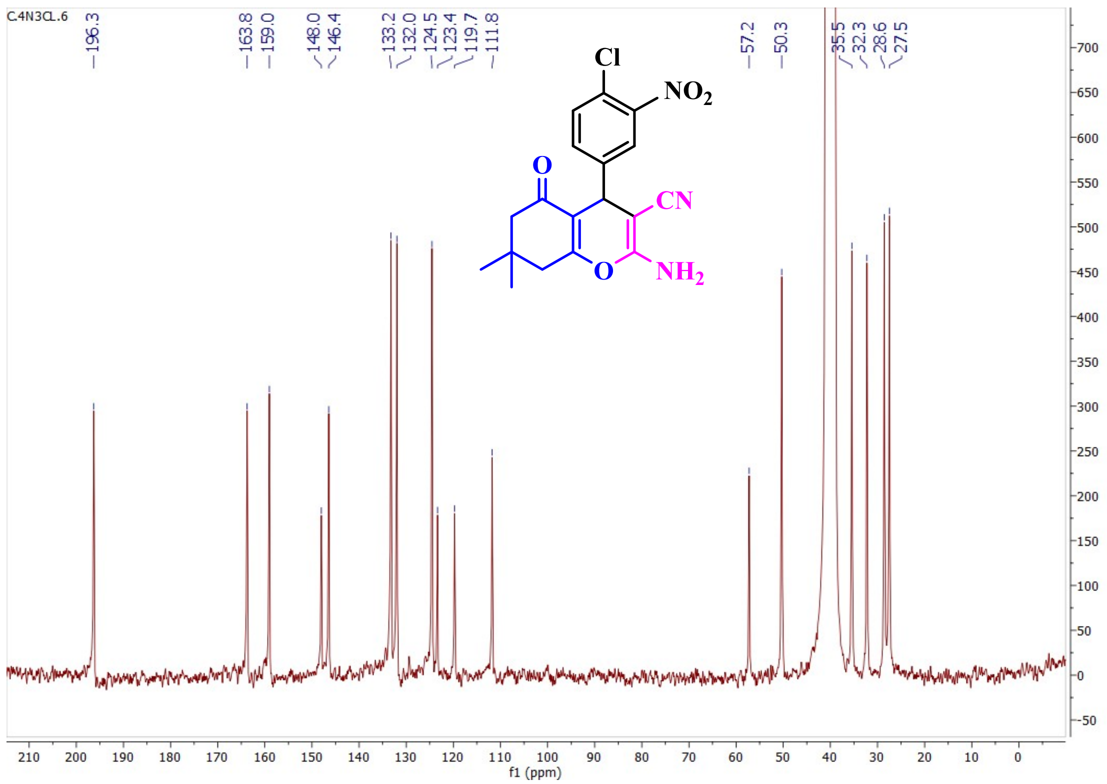

Figure 8. Spectrum ^{13}CNMR (63 MHz) of 2 -amino-4-(4-chloro-3-nitrophenyl)-7,7-dimethyl-5-oxo-5,6,7,8-tetrahydro-4H-chromene-3-carbonitrile in DMSO solvent

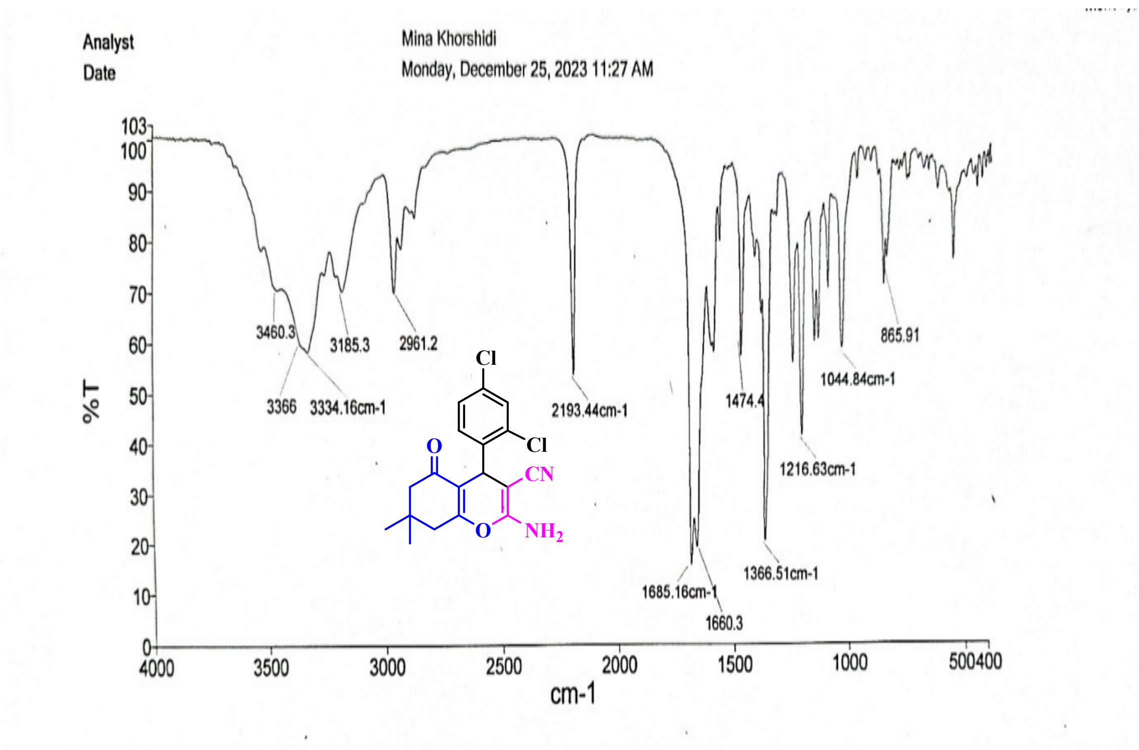

Figure 9. Spectrum FTIR of 2-amino-4-(2,4-dichlorophenyl)-7,7-dimethyl-5-oxo-5,6,7,8-tetrahydro-4H-chromene-3-carbonitrile

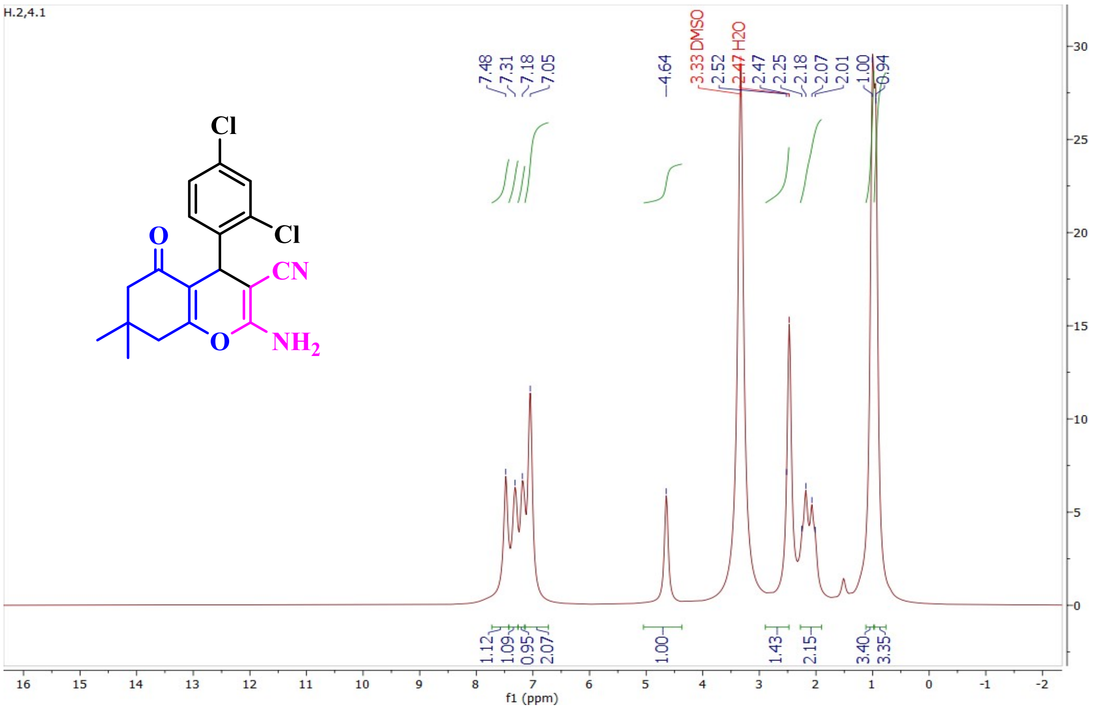

Figure 10. Spectrum ^{1}H NMR (250 MHz) of 2-amino-4-(2,4-dichlorophenyl)-7,7-dimethyl-5-oxo-5,6,7,8-tetrahydro-4H-chromene-3-carbonitrile in DMSO solvent

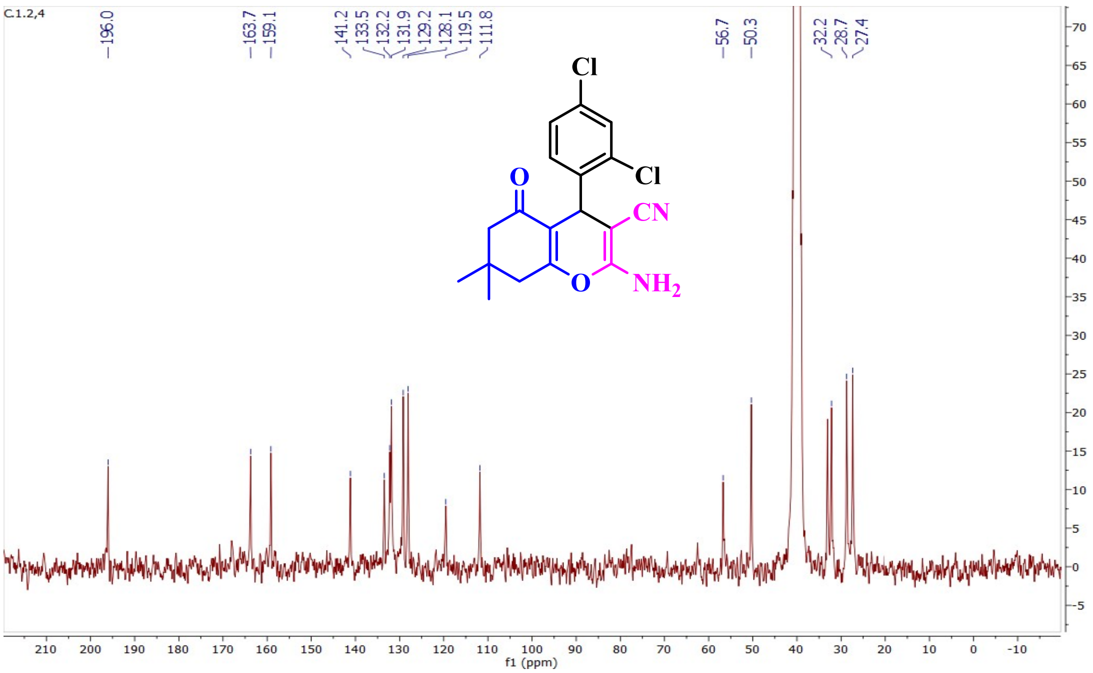

Figure 11. Spectrum ^{13}CNMR (63 MHz) of 2-amino-4-(2,4-dichlorophenyl)-7,7-dimethyl-5-oxo-5,6,7,8-tetrahydro-4H-chromene-3-carbonitrile in DMSO solvent

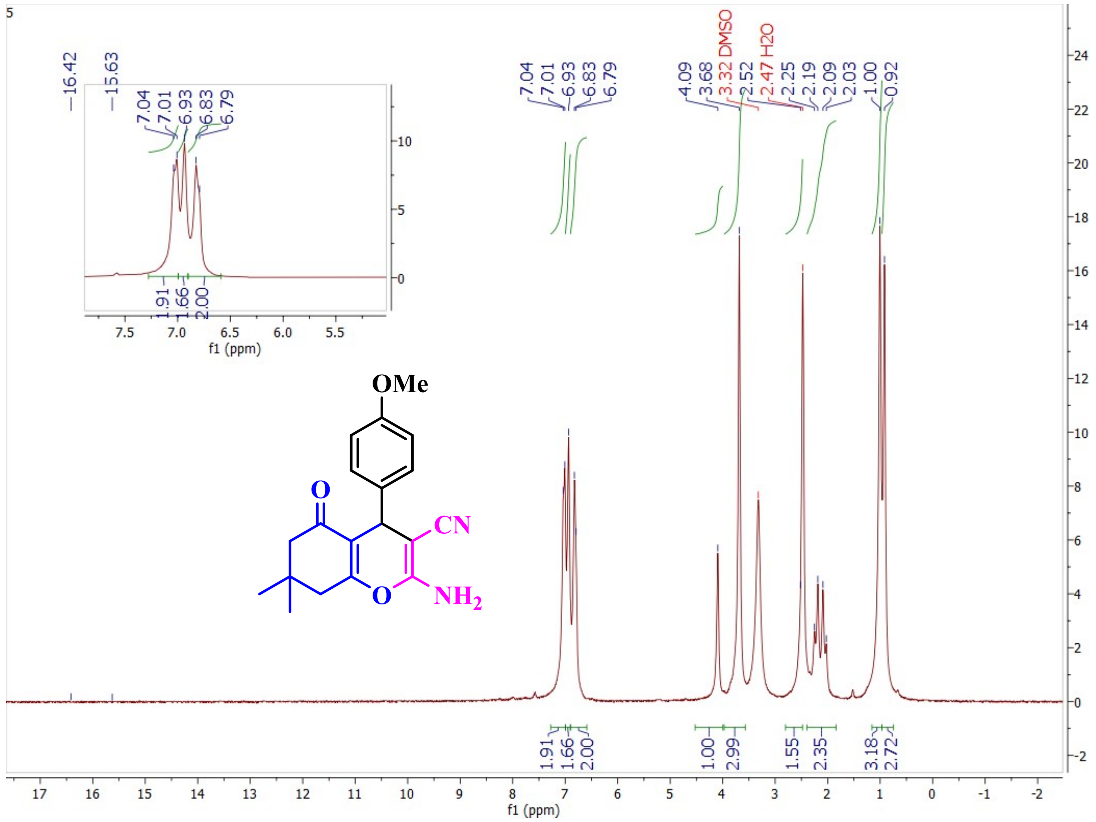

Figure 12. Spectrum ^{1}H NMR (250 MHz) of 2-amino-4-(4-methoxyphenyl)-7,7-dimethyl-5-oxo-5,6,7,8-tetrahydro-4H-chromene-3-carbonitrile in DMSO solvent

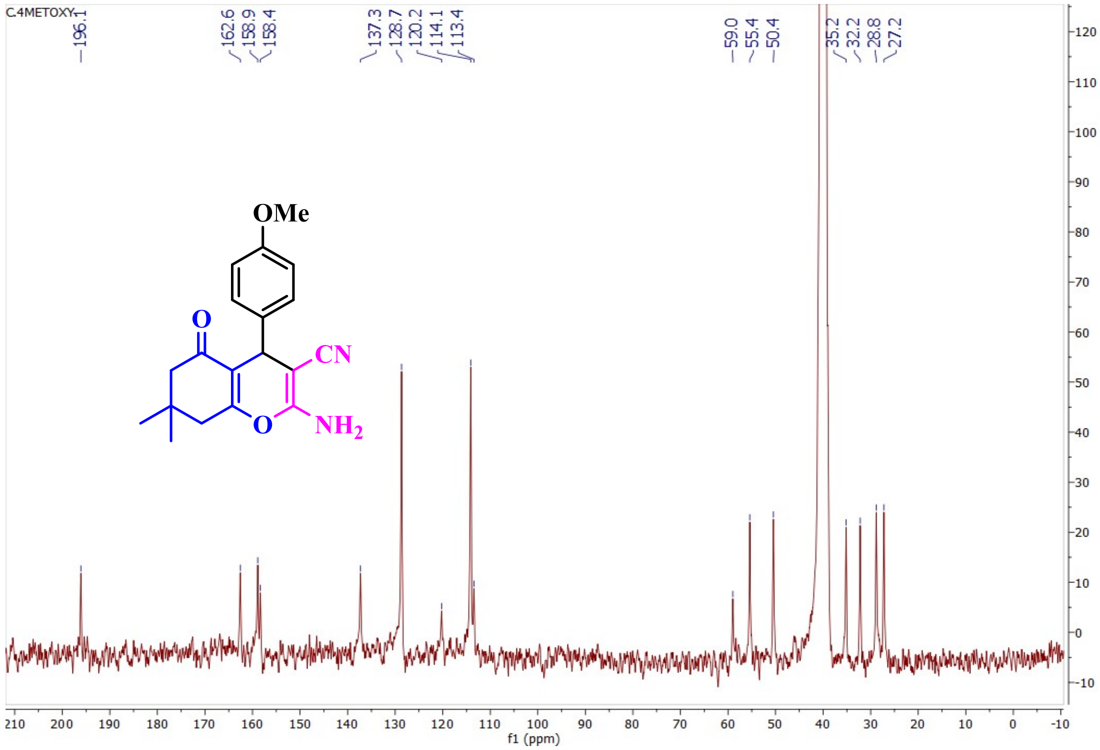

Figure 13. Spectrum ^{13}CNMR (63 MHz) of 2-amino-4-(4-methoxyphenyl)-7,7-dimethyl-5-oxo-5,6,7,8-tetrahydro-4H-chromene-3-carbonitrile in DMSO solvent

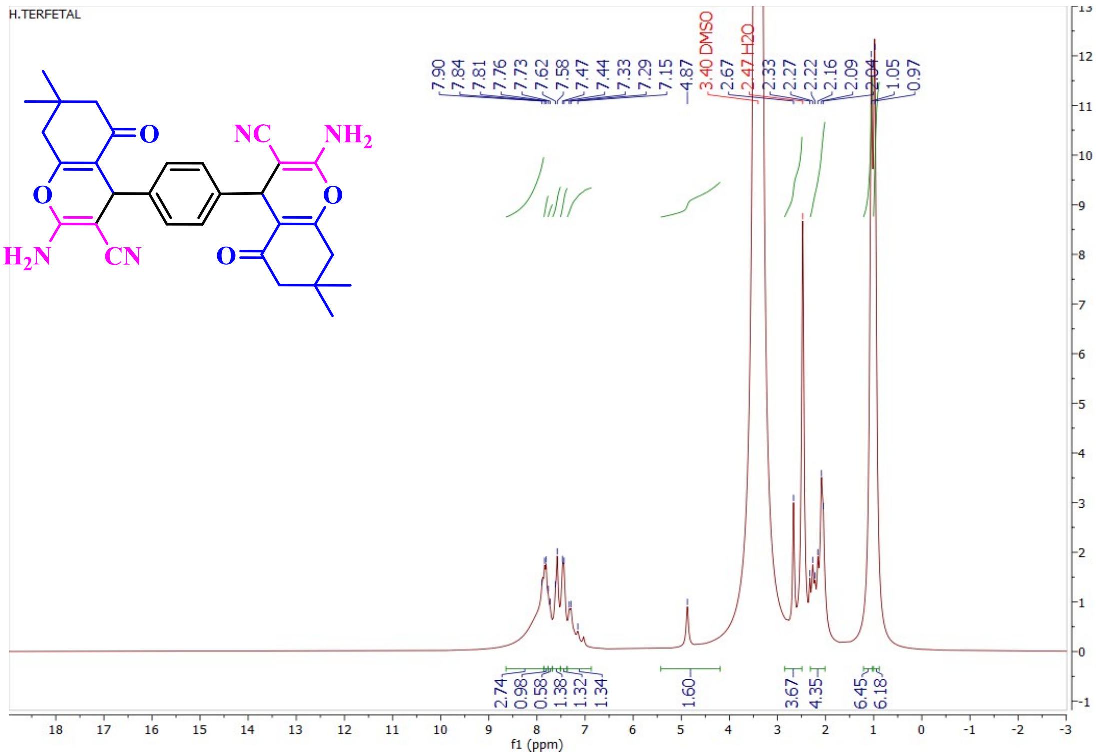

Figure 14. Spectrum ^{1}H NMR (250 MHz) of 4,4'-(1,4-phenylene)bis(2-amino-7,7-dimethyl-5-oxo-5,6,7,8-tetrahydro-4H-chromene-3-carbonitrile) in DMSO solvent

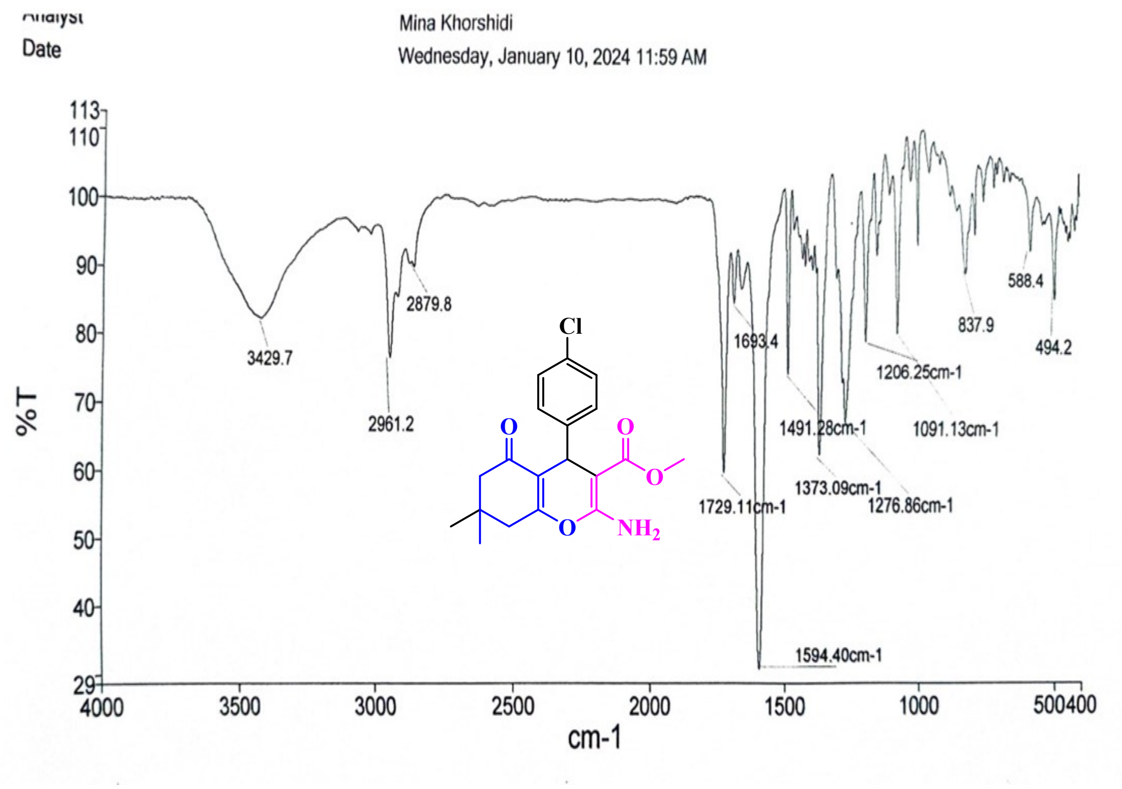

Figure 15. Spectrum FTIR of methyl 2-amino-4-(4-chlorophenyl)-7,7-dimethyl-5-oxo-5,6,7,8-tetrahydro-4H-chromene-3-carboxylate

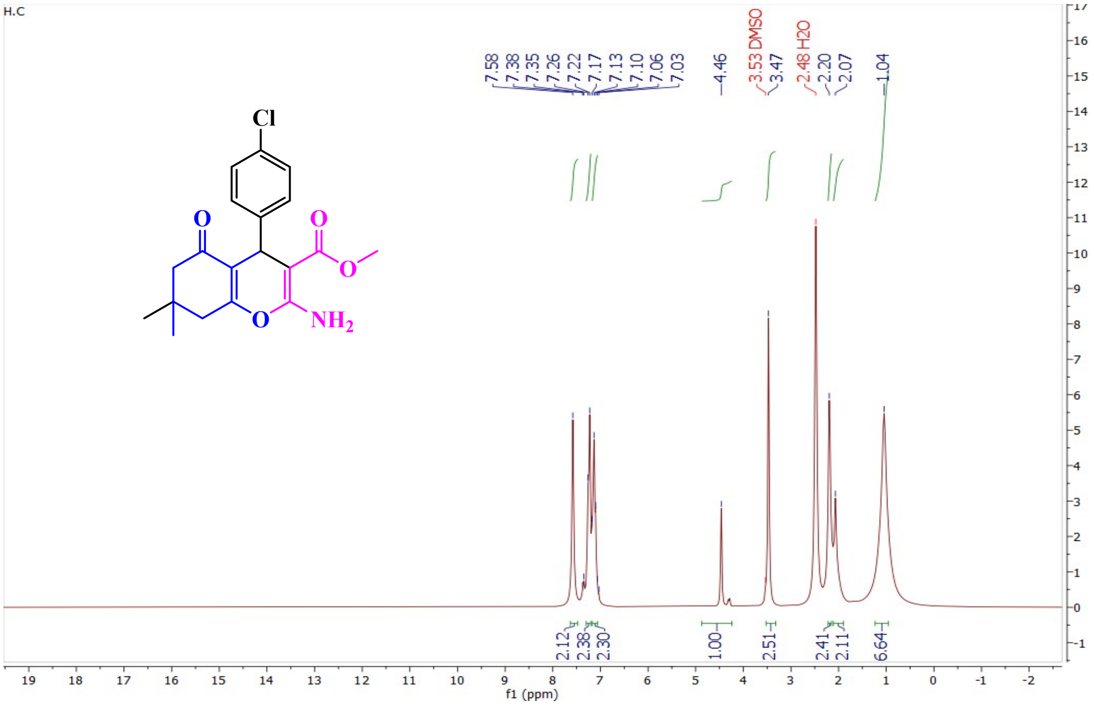

Figure 16. Spectrum ^{1}H NMR (250 MHz) of methyl 2-amino-4-(4-chlorophenyl)-7,7-dimethyl-5-oxo-5,6,7,8-tetrahydro-4H-chromene-3-carboxylate in DMSO solvent

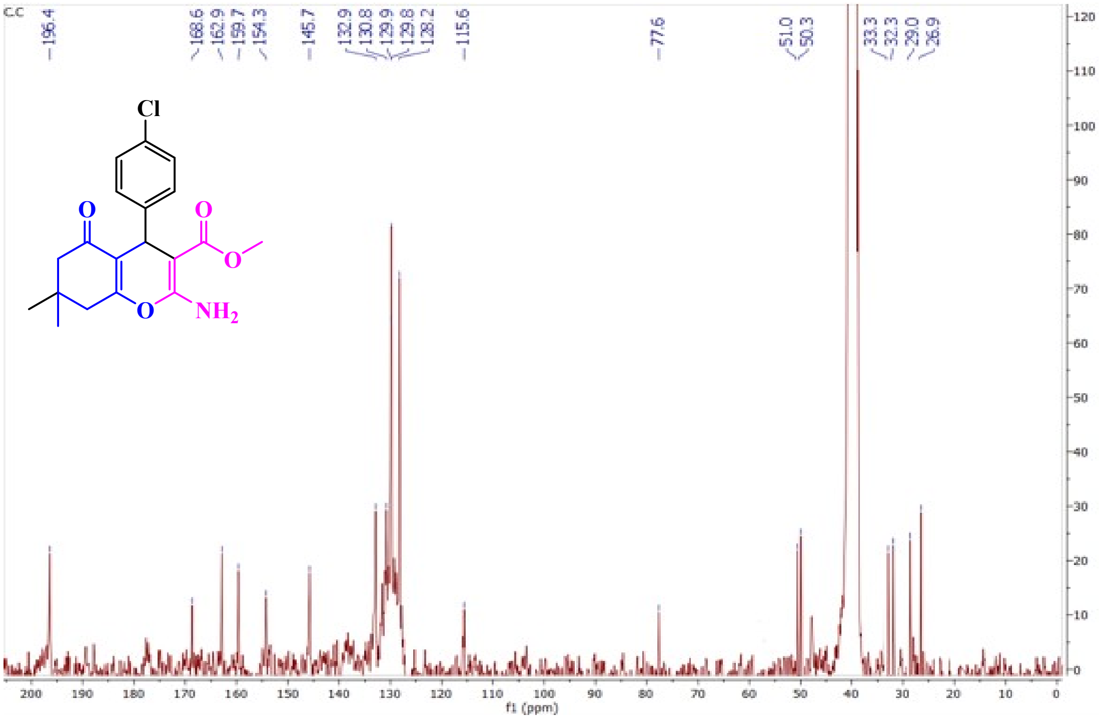

Figure 17. Spectrum ^{13}CNMR (63 MHz) of methyl 2-amino-4-(4-chlorophenyl)-7,7-dimethyl-5-oxo-5,6,7,8-tetrahydro-4H-chromene-3-carboxylate in DMSO solvent

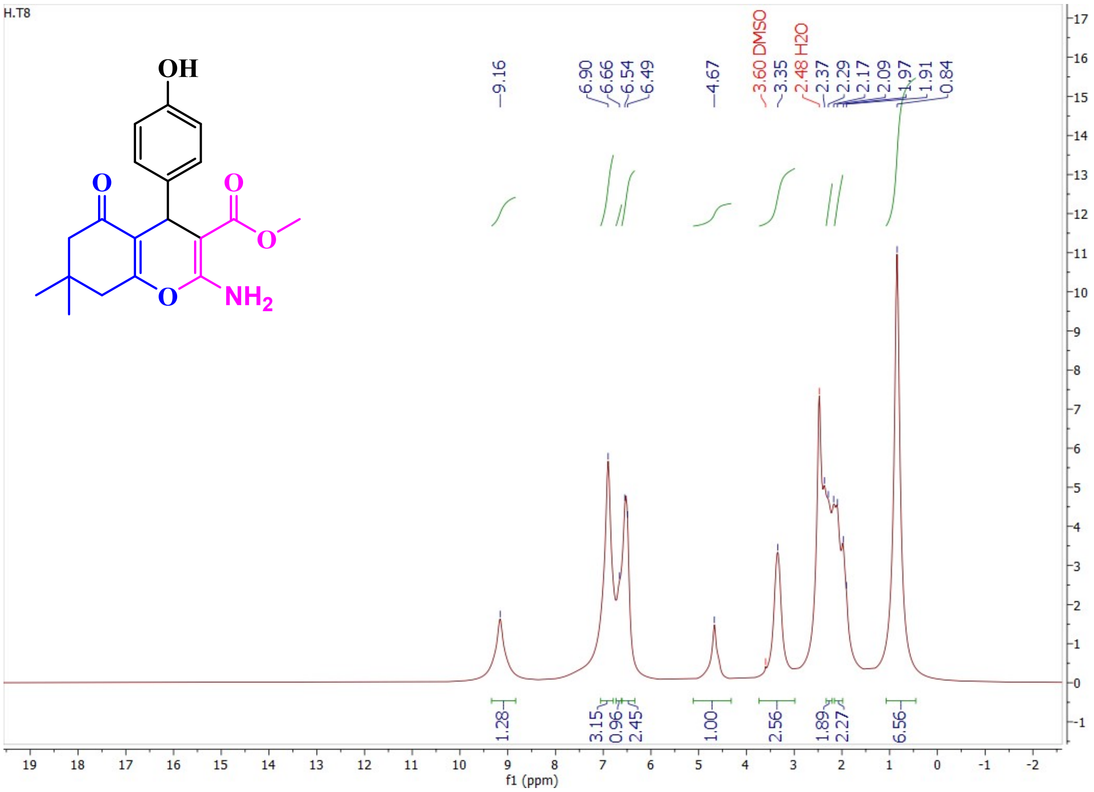

Figure 18. Spectrum ^{1}H NMR (250 MHz) of methyl 2-amino-4-(4-hydroxyphenyl)-7,7-dimethyl-5-oxo-5,6,7,8-tetrahydro-4H-chromene-3-carboxylate in DMSO solvent

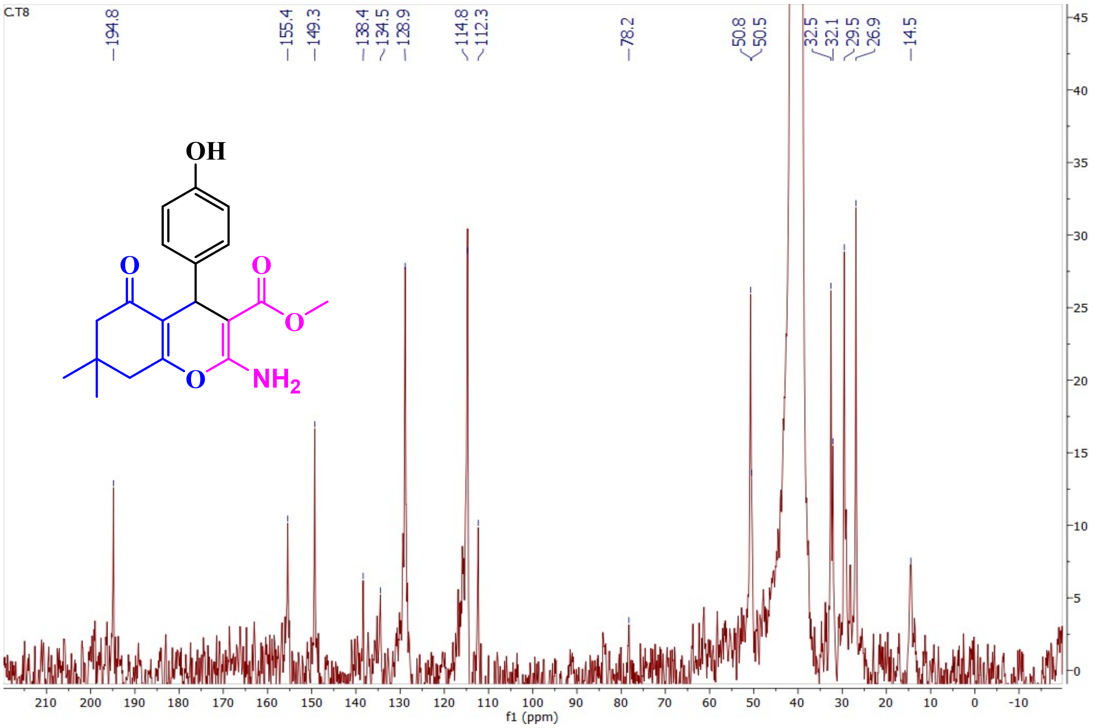

Figure 19. Spectrum ^{13}C NMR (63 MHz) of methyl 2-amino-4-(4-hydroxyphenyl)-7,7-dimethyl-5-oxo-5,6,7,8-tetrahydro-4H-chromene-3-carboxylate in DMSO solvent

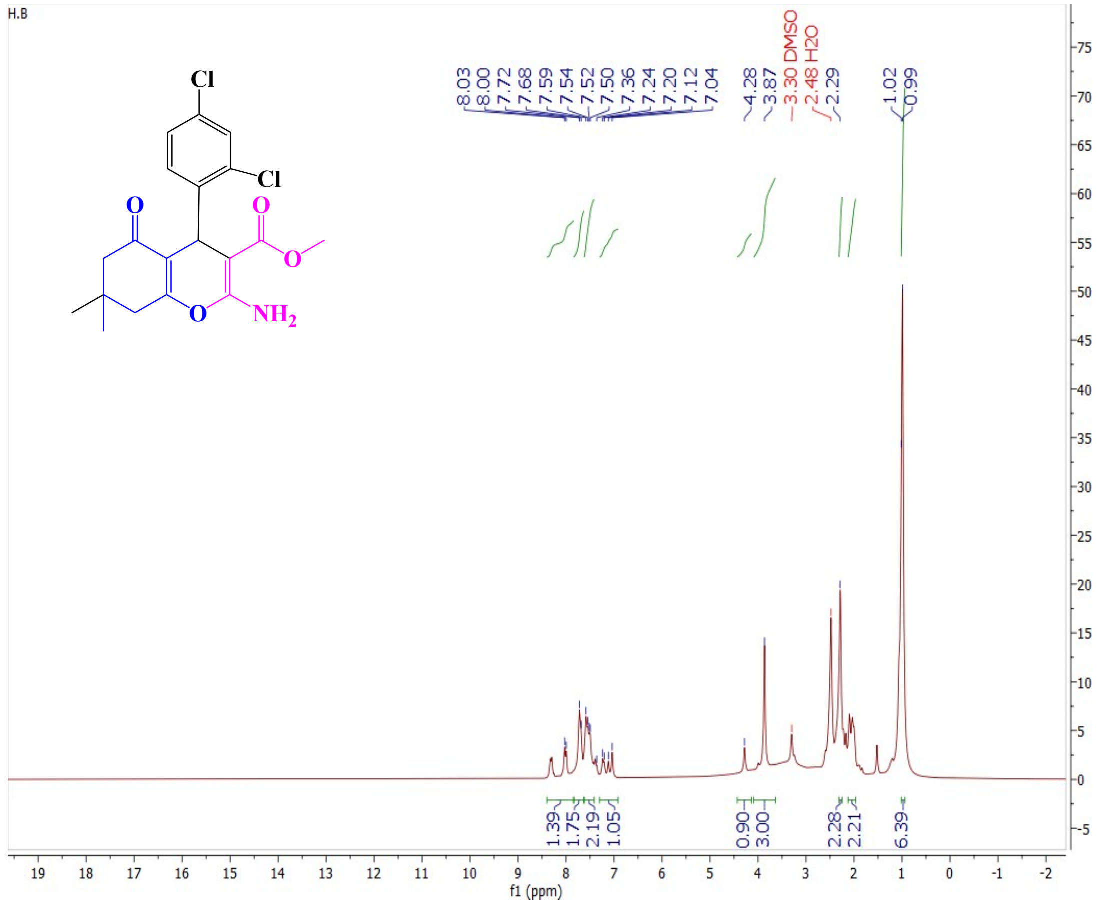

Figure 20. Spectrum ^{1}H NMR (250 MHz) of methyl 2-amino-4-(2,4-dichlorophenyl)-7,7-dimethyl-5-oxo-5,6,7,8-tetrahydro-4H-chromene-3-carboxylate in DMSO solvent

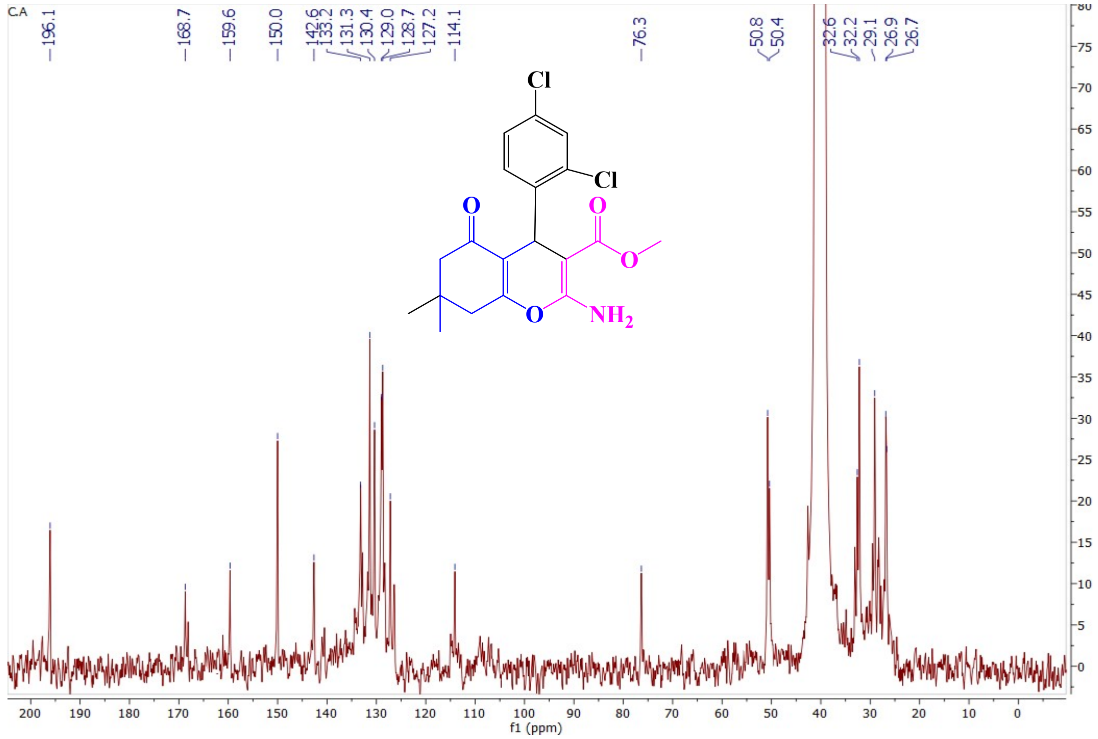

Figure 21. Spectrum ^{13}C NMR (63 MHz) of methyl 2-amino-4-(2,4-dichlorophenyl)-7,7-dimethyl-5-oxo-5,6,7,8-tetrahydro-4H-chromene-3-carboxylate in DMSO solvent

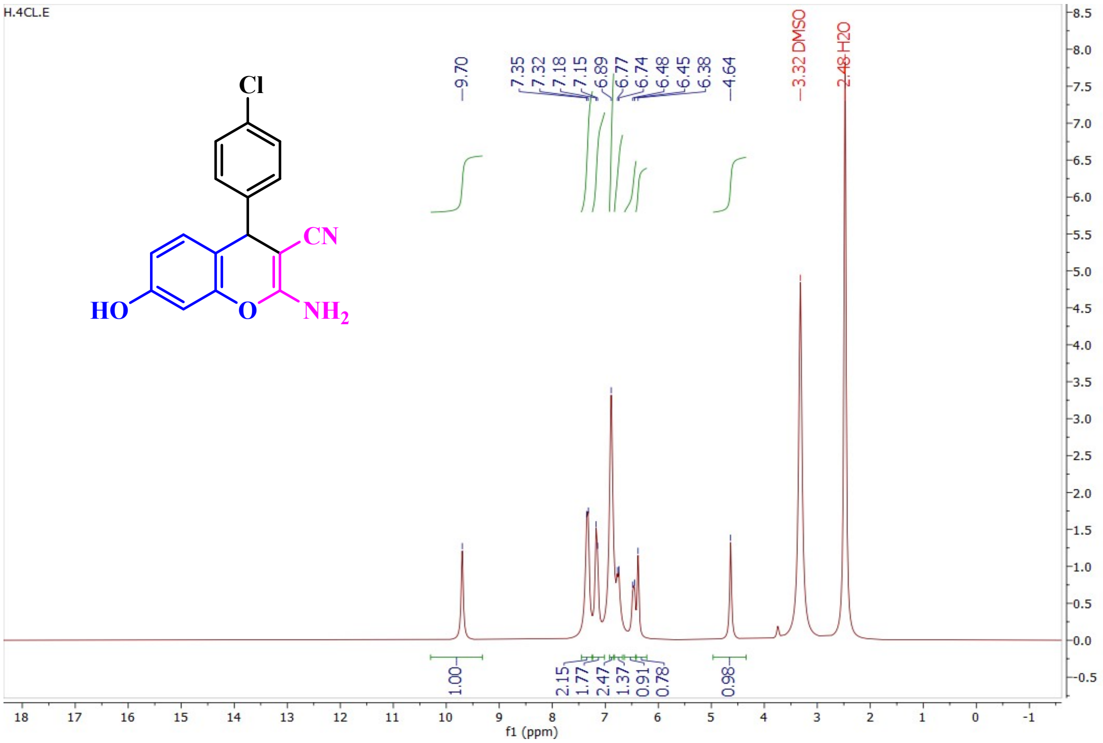

Figure 22. Spectrum ^{1}H NMR (250 MHz) of 2-amino-4-(4-chlorophenyl)-7-hydroxy-4H-chromene-3-carbonitrile in DMSO solvent

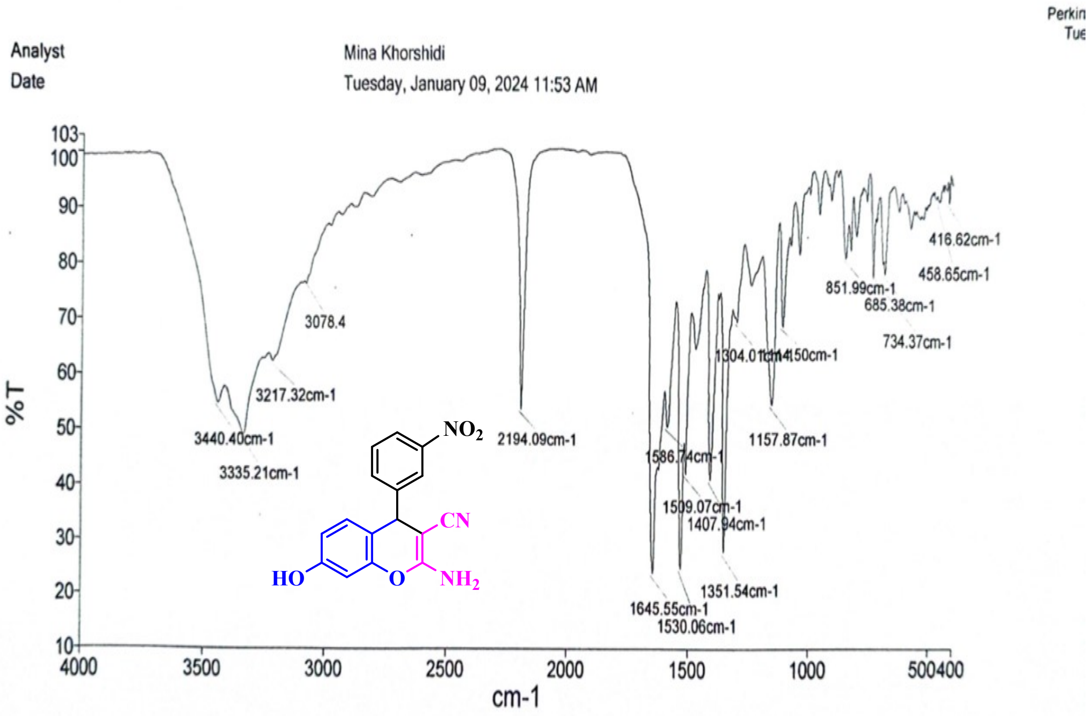

Figure 23. Spectrum FTIR of 2-amino-7-hydroxy-4-(3-nitrophenyl)-4H-chromene-3-carbonitrile

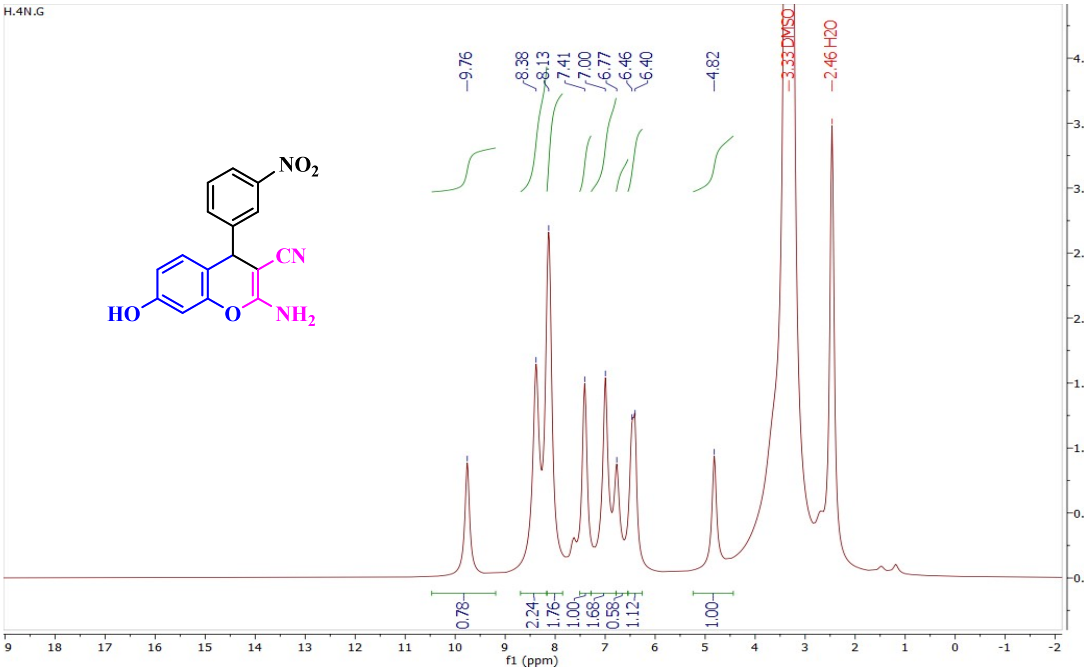

Figure 24. Spectrum ^{1}H NMR (250 MHz) of 2-amino-7-hydroxy-4-(3-nitrophenyl)-4H-chromene-3-carbonitrile in DMSO solvent

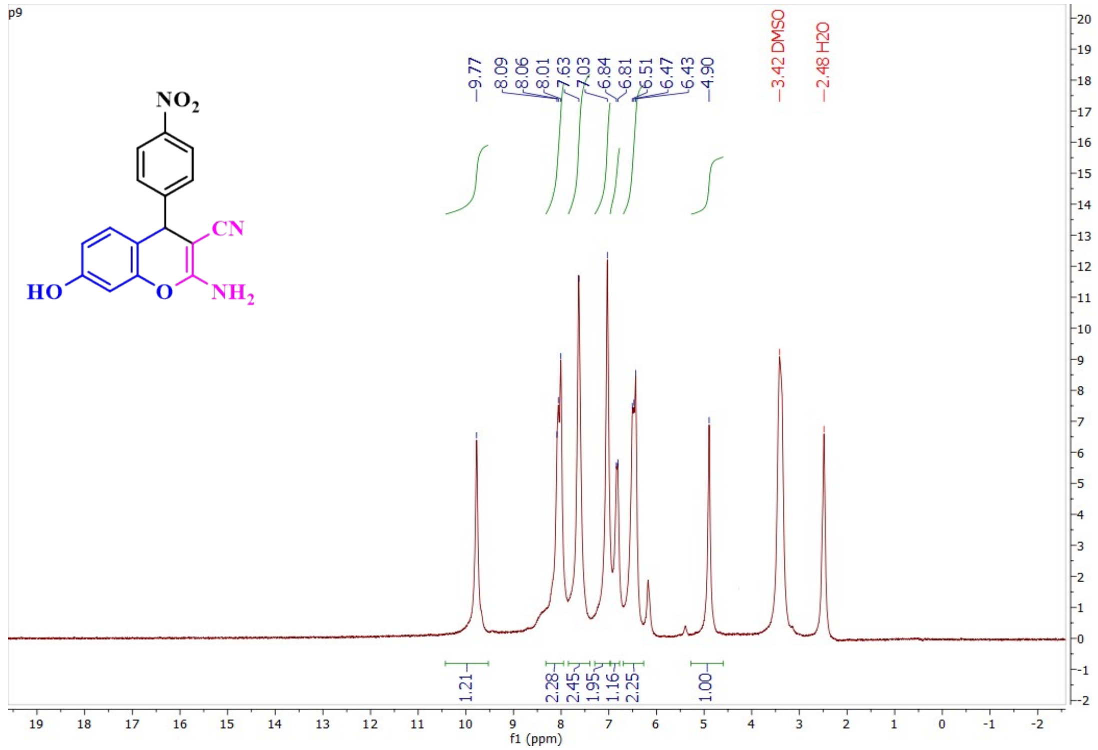

Figure 25. Spectrum ^{1}H NMR (250 MHz) of 2-amino-7-hydroxy-4-(4-nitrophenyl)-4H-chromene-3-carbonitrile in DMSO solvent

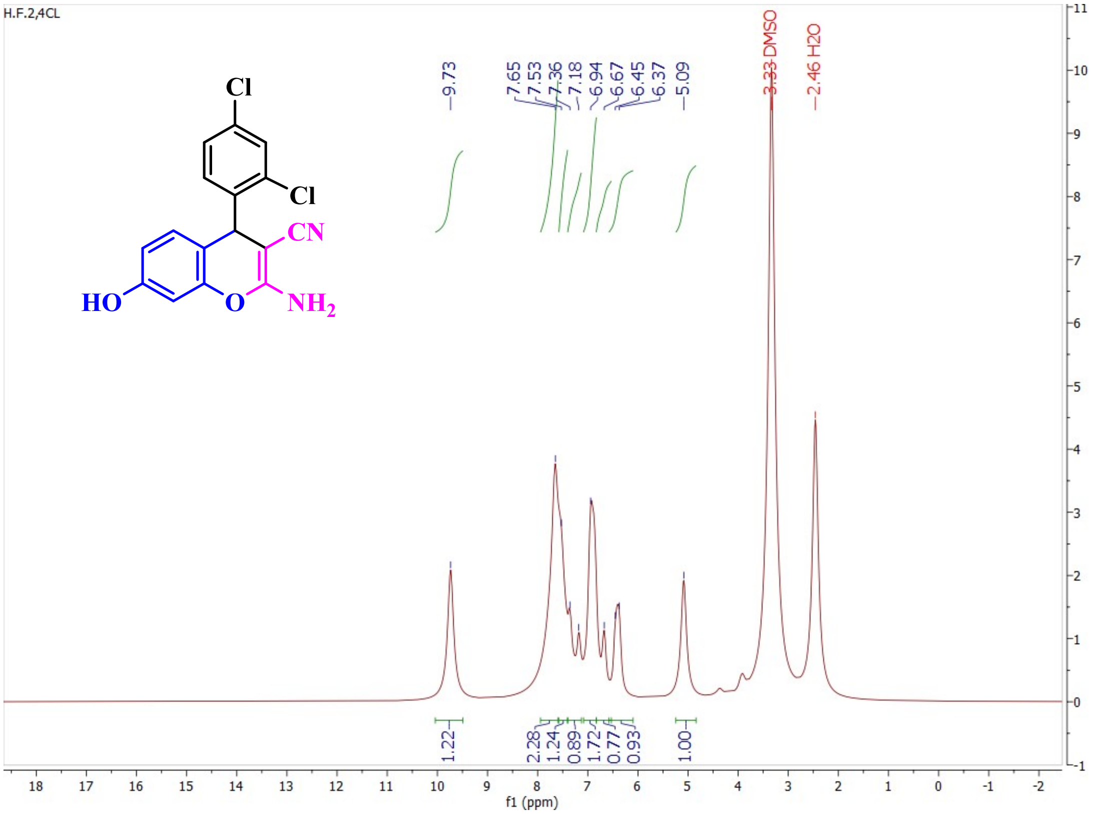

Figure 26. Spectrum ^{1}H NMR (250 MHz) of 2-amino-4-(2,4-dichlorophenyl)-7-hydroxy-4H-chromene-3-carbonitrile in DMSO solvent
